# Effect of curcumin-pipeine supplementation on clinical status, mortality rate, oxidative stress, and inflammatory markers in critically ill ICU patients with COVID-19: a structured summary of a study protocol for a randomized controlled trial

**DOI:** 10.1186/s13063-021-05372-9

**Published:** 2021-07-06

**Authors:** Gholamreza Askari, Babak Alikiaii, Davood Soleimani, Amirhossein Sahebkar, Mahdiye Mirjalili, Awat Feizi, Bijan Iraj, Mohammad Bagherniya

**Affiliations:** 1 Food Security Research Center, Isfahan University of Medical Sciences, PO Box: 00983117922110, Isfahan, Iran; 2 Anesthesia and Critical Care Research Center, Isfahan University of Medical Sciences, Isfahan, Iran; 3 Department of Community Nutrition, School of Nutrition and Food Science, Isfahan University of Medical Sciences, Isfahan, Iran; 4 Research Center of Oils and Fats, Kermanshah University of Medical Sciences, Kermanshah, Iran; 5 Applied Biomedical Research Center, Mashhad University of Medical Sciences, Mashhad, Iran; 6 Biotechnology Research Center, Pharmaceutical Technology Institute, Mashhad University of Medical Sciences, Mashhad, Iran; 7 School of Pharmacy, Mashhad University of Medical Sciences, Mashhad, Iran; 8 Department of Biostatistics and Epidemiology, School of Health, Isfahan University of Medical Sciences, Isfahan, Iran; 9 Isfahan Endocrine and Metabolism Research Center, Isfahan University of Medical Sciences, Isfahan, Iran

**Keywords:** COVID-19, Coronavirus, Curcumin, ICU Patients, Randomised controlled trial, Protocol

## Abstract

**Objectives:**

This study aims to investigate the efficacy of curcumin-piperine co-supplementation on oxidative stress factors, clinical symptoms, and mortality rate in patients with coronavirus (COVID-19) admitted to the intensive care unit (ICU).

**Trial design:**

This study is a randomized, placebo-controlled, double-blind, parallel-arm clinical trial.

**Participants:**

The study participants will be recruited from patients admitted to the ICU of Al-Zahra hospital with a definitive diagnosis of COVID-19. The inclusion criteria are aged between 20 and 75 years, confirmation of COVID-19 based on the PCR test, and admitted to the ICU. The exclusion criteria include the present use of parenteral nutrition support, a history of underlying diseases such as congenital disorders, immune diseases, renal and hepatic insufficiency, and pancreatitis, a history of sensitivity to herbal remedies such as turmeric and pepper, and regular use of anticoagulant drugs such as warfarin. This study will be performed in the Al-Zahra hospital, an academic hospital affiliated with Isfahan University of Medical Sciences, Isfahan, Iran.

**Intervention and comparator:**

Sixty eligible patients will be randomly assigned, in a 1:1 ratio, to receive curcumin-piperine capsules (three capsules/day; each capsules containing 500 mg curcumin plus 5 mg piperine; in total 1500 mg curcumin and 15 mg piperine/daily) for seven days (n=30) or matching placebo capsules (three capsules/day; each capsules containing 505 mg maltodextrin; totally 1515 mg, maltodextrin/ daily) for same duration (n=30). Capsules will be administered after oral or enteral feeding at 9, 15 and 21 o’clock.

**Main outcomes:**

The primary outcome is the time from initiation of supplementation (curcumin-piperine or placebo) to normalization of fever, respiratory rate, and blood oxygen saturation. The secondary outcomes are the mortality rate, length of stay in ICU, temperature, levels of blood oxygen saturation, ventilator dependency, respiratory rate, levels of C-reactive protein (CRP), erythrocyte sedimentation rate (ESR), levels of liver markers (ALT, AST, LDH), and levels of kidney function markers (BUN, Creatinine).

**Follow up:**

All of the parameters will be assessed at baseline and end of the study (7 days intervention). In addition, the rate of mortality will be collected after 4 weeks (28 days’ mortality in the ICU, 4 weeks follow up).

**Randomisation:**

Eligible patients will be randomly assigned to either the intervention group (Curcumin-piperine) or the control group (Placebo). Randomization sequences will be generated using an electronic table of random numbers to allocate the included participants into either control or intervention groups (in a 1:1 ratio) using the stratified block randomization method. Stratification was conducted according to sex (male and female), with a block size of four. The allocation sequences will be prepared by an independent statistician and will be kept inside sealed, opaque, and consecutively numbered envelopes. Participants, investigators, nurses, and physicians will be unaware of the trial-group assignment.

**Blinding (masking):**

This study is a double-blind clinical trial (participants, investigators, nurses, and physicians). The curcumin-piperine and placebo supplements will be similar in the terms of texture, taste, color, odor, and weight. Both tablets will be provided in containers that are completely identical in weight, shape, labeling, and packaging. All participants, investigators, nurses, and physicians will be unaware of the trial-group assignment.

**Numbers to be randomised (sample size):**

The sample size is estimated at 60 participants, including 30 patients in the intervention group and 30 patients in the placebo group.

**Trial Status:**

The protocol is Version 2, registered on May 13, 2021. Recruitment began May 20, 2021, and is anticipated to be completed by September 20, 2021.

**Trial registration:**

This trial has been registered in Iranian Registry of Clinical Trials (IRCT) with the title of “Evaluation of the effect of curcumin-piperine supplementation in patients with coronavirus admitted to the intensive care unit (ICU): a double-blind clinical trial study”. IRCT registration number is IRCT20121216011763N52. The registration date was May 13, 2021.

**Full Protocol:**

The full protocol is attached as an additional file, accessible from the Trials website (File 1). In the interest of expediting the dissemination of this material, the familiar formatting has been eliminated; this Letter serves as a summary of the key elements of the full protocol.

**Supplementary Information:**

The online version contains supplementary material available at 10.1186/s13063-021-05372-9.

## Supplementary Information

**Additional file 1.** Full study protocol.

## Data Availability

The final dataset of the trial will be available upon request from the primary investigator via e-mail at bagherniya@nutr.mui.ac.ir, after obtaining the permission of the Regional Ethics Committee.

