# Supplementary material for: Effect of curcumin-pipeine supplementation on clinical status, mortality rate, oxidative stress, and inflammatory markers in critically ill ICU patients with COVID-19: a structured summary of a study protocol for a randomized controlled trial
Source: Trials. 2021 Jul 6;22:434. doi: 10.1186/s13063-021-05372-9 (PMC8258487; doi:10.1186/s13063-021-05372-9)
Supplement: Supplementary file 1 — Additional file 1. Full study protocol. [file 13063_2021_5372_MOESM1_ESM.docx]

| 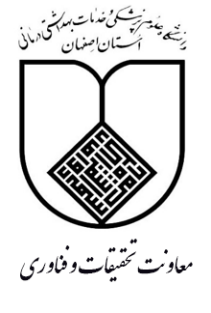 | 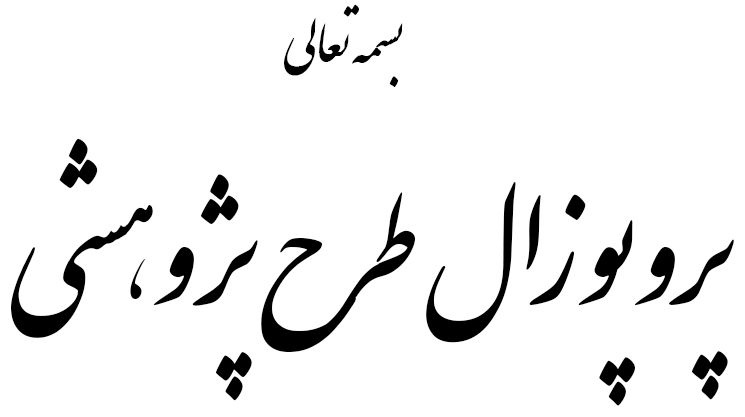  (RES-02) |  |
| --- | --- | --- |

**الف) چکیده**

| **مجری اصلی** | | | |
| --- | --- | --- | --- |
| **غلامرضا عسکری** | | | |
| **همکاران طرح** | | | |
| **در مورد طرح­های تحقیقاتی، نام و نام خانوادگی همکاران علمی و اجرایی نوشته شود.**  **در مورد پایان­نامه­های تحصیلی جدول زیر تکمیل شود و در مورد سایر طرح­ها این جدول حذف شود.**   \| دانشجو \| \| \| \| \| --- \| --- \| --- \| --- \| \| **-** \| **-** \| **-** \| **-** \| \| استاد/استادان مشاور \| \| \| \| \| **-** \| **-** \| **-** \| **-** \| \| سایر همکاران \| \| \| \| \| بابک علی کیایی \| دانشیار آی سی یو و بیهوشی، گروه بیهوشی \| دانشگاه علوم پزشکی اصفهان \| مجری \| \| محمد باقرنیا \| استادیار تغذیه، گروه تغذیه جامعه \| دانشگاه علوم پزشکی اصفهان \| مجری \| \| بیژن ایرج \| دانشیار (فوق تخصص) غدد، گروه غدد \| دانشگاه علوم پزشکی اصفهان \| مجری \| \| آوات فیضی \| استاد، گروه آمار و اپیدمیولوژی \| دانشگاه علوم پزشکی اصفهان \| همکار \| | | | |
| **عنوان طرح** | | | |
| **بررسی اثر مکملیاری با کورکومین-پیپرین در بیماران مبتلا به ویروس کرونا بستری در بخش مراقبت های ویژه (ICU): یک مطالعه کارآزمایی بالینی دو سو کور** | | | |
| **Evaluation of the effect of curcumin-piperine supplementation in patients with coronavirus admitted to the intensive care unit (ICU): a double-blind clinical trial study** | | | |
| **چکیده** | | | |
| **خلاصه بیان مساله و ضرورت اجرا (حداکثر 300 کلمه)**  ویروس کرونا^[[1]](#footnote-1)^ شامل خانواده بزرگی از ویروس هاست که از بیماری سرماخوردگی تا بیماری های شدیدتر مانند سندرم تنفسی خاورمیانه (MERS-CoV)^[[2]](#footnote-2)^ و سندرم حاد تنفسی^[[3]](#footnote-3)^ (SARS-CoV) را شامل می شود. ویروس کرونا جدید^[[4]](#footnote-4)^ (nCoV) نوعی ویروس جدید است که قبلاً در انسان مشخص نشده است. ویروس کرونا عامل ایجاد بیماری های تنفسی، دستگاه گوارش، کبدی و سیستم عصبی مرکزی در انسان، دام و حیوانات وحشی است. خفاش ها به عنوان حامل طبیعی انواع ویروس کرونا معرفی شده اند. علائم شایع عفونت در این بیماری، شامل علائم تنفسی، تب، سرفه، تنگی نفس و مشکلات تنفسی است. در موارد شدیدتر، عفونت می تواند باعث ذات الریه، سندرم حاد تنفسی شدید، نارسایی کلیه و حتی مرگ شود. یکی از مهمترین عوارض ویروس کرونا سندرم حاد تنفسی حاد (SARS) می باشد. SARS در اواخر سال 2002 از جنوب چین سرچشمه گرفته و مرگ و میر و عوارض بالایی دارد. طی یک دوره شش ماهه از اواخر سال 2002 ، این بیماری بیش از 8000 نفر را مبتلا کرده و نزدیک به 800 نفر را کشته است. این بیماری تهدیدی جدید برای سلامت انسان ها محسوب می شود و یک چالش برای توسعه و تجویز داروهای ضد ویروسی است. SARS توسط یک ویروس کرونای جدید مرتبط با SARS ایجاد می شود (بیماری همه گیر پنومونی آتیپیک) که توسط یک شبکه آزمایشگاهی جهانی تحت نظارت سازمان بهداشت جهانی (WHO) شناسایی شده است. این ویروس ها معمولا به عنوان بیماری تنفسی و دستگاه گوارش در انسان و حیوانات اهلی شناخته می شوند. کرونا ویروس 19 (COVID-19)، ویروس جدیدی بوده و به خانواده ویروس‌هایی مانند SARS (سندرم تنفسی حاد) و سایر سرماخوردگی‌های معمولی مرتبط است. بر اساس مطالعات اخیر، ویژگی اصلی ویروس کرونای جدید سندرم حاد تنفسی است و در واقع SARS-CoV-2 (سندرم حاد تنفسی حاد نوع 2) می باشد که سازمان بهداشت جهانی آن را COVID-19 نامیده است. اگرچه اتیولوژی این بیماری هنوز به طور کامل شناخته شده نیست اما افزایش التهاب ریه ها یکی از عواملی است که بصورت منفی بر بیماری اثر می گذارد. لذا در بعضی موارد برای کاهش التهاب و کنترل بهتر بیماری از داروهای ضد التهابی استروییدی استفاده گردیده که مشخص شده این امر موجب کاهش توان سیستم ایمنی بدن و در نتیجه کاهش مقاومت در برابر بیماری و اثرات نامطلوب بسیاری برای فرد می گردد. با توجه به اپیدمی شدن ناگهانی این بیماری، تاکنون راهکار درمانی مناسبی برای آن معرفی نشده است.  یکی از مواردی که می تواند موجب کاهش التهاب و بهبود وضعیت این بیماران گردد گیاهان دارویی است که مطالعات مختلف نشان داده اند که این گیاهان اثرات ضدالتهابی، ضد استرس اکسیداتیو، ضد باکتریایی و ضد عفونت دارند، در عین حال معمولا عارضه خاصی ندارند و میتوانند بعنوان طب مکمل برای بهبود وضعیت بیماری مورد استفاده قرار گیرد. از جمله این گیاهان کورکومین است. کورکومین دارای فعالیت های آنتی اکسیدانی، ضد التهابی، تعدیل کننده سیستم ایمنی، اثرات مطلوب روی مقاومت به انسولین، فشار خون، چربی خون، آنزیم های کبدی و بسیاری دیگر از شاخص های مرتبط با سلامتی انسان می باشد و می تواند یک ماده طبیعی بالقوه برای پیشگیری و یا درمان برخی از بیماری ها باشد. یافته های مطالعات قبل بیانگر اثرات ضد میکروبی، ضد قارچی، ضد ویروس و ضد پنومونی بوده است. هم چنین مطالعات پره کلینیکال اخیر نشان داده اند که کورکومین می تواند بعنوان یک ماده گیاهی جدید اثرات مطلوبی بر آنفولانزا داشته باشد بطوریکه باعث کاهش تولید مثل ویروس، کاهش التهاب ریه ها و پنومونی میگردد. همچنین اثرات مطلوب کورکومین بر روی بیماری های ریوی به خصوص آسیب و فیبروز ریه ها و کاهش التهاب ریه ها که در اثر بیماری های مختلف و همچنین پرتودرمانی ، داروهای شیمی درمانی و سموم بوجود می آید را نشان داده اند.  نکته حائز اهمیت درباره کورکومین اینست که علیرغم پتانسیل درمانی بالا، به علت حلالیت آبی ضعیفی که دارد و همچنین زیست دسترسی پایین و تخریب سریع آن برای استفاده در اهداف پزشکی دارای محدودیت می باشد. با این حال، برای بهبود پارامترهای مرتبط با بالینی آن، فرمولاسیون کورکومین همراه با پیپرین به عنوان یک جایگزین جدید برای روش های درمانی برتر خود در حال ظهور است. پیپرین از طریق افزایش زیست فراهمی کورکومین و کاهش کونژوگاسیون کبدی آن با گلوکورونیک اسید و در نتیجه کاهش دفع ادراری آن این محدودیت ها را برطرف می سازد.  با توجه به عدم درمان مناسب و قطعی برای انواع بیماری های ویروسی مانند آنفولانزا و کرونا و عوارض و پیامدهای بیشمار این بیماری ها که منجر به تحمیل خسارت های بیشمار به سیستم بهداشتی کشورها می گردد بنظر می رسد انجام مداخلات جهت یافتن راهکاری مناسب و مقرون به صرفه اهمیت فوق العاده ای دارد.  با توجه به اثرات بسیار مناسب کورکومین بر جنبه های مختلف سلامتی انسان و اثرات مطلوب آن بر بیماری های عفونی مانند آنفولانزا و پنومونی در مطالعات پایه، بنظر می رسد استفاده از مکمل کورکومین پیپرین بعنوان یک مکمل غذایی گیاهی، طبیعی، ارزان قیمت، در دسترس و بدون عارضه در بیماران مبتلا به ویروس کرونا می تواند باعث بهبود وضعیت این بیماران گردد. لذا هدف از مطالعه حاضر بررسی اثر مکمل یاری با کورکومین پیپرین (سه کپسول 500 میلی گرمی کورکومین + 5 میلی گرم پیپرین در روز برای دو هفته) بر روی بیماران مبتلا به ویروس کرونا بستری در بخش مراقبت های ویژه طی یک مطالعه کارآزمایی بالینی دو سو کور می باشد. امید است نتایج این مطالعه بتواند باعث بهبود وضعیت سلامتی بیماران مبتلا به ویروس کرونا گردد. | | | |
| **خلاصه روش اجرا (حداکثر 300 کلمه)**  این مطالعه به صورت یک کارآزمایی بالینی تصادفی دوسوکور به مدت 2 هفته (14 روز) بر روی 50 فرد مبتلا به ویروس کرونا انجام خواهد شد. برای این منظور 50 بیمار بستری در ICU که ابتلای آنها به COVID19 بر اساس PCR تشخیص داده شده است به طور تصادفی به دو گروه تقسیم خواهند شد (25 نفر بیمار در گروه مداخله و 25 نفر در گروه کنترل). گروه مداخله؛ روزانه سه کپسول (هر کپسول حاوی 500 میلی گرم کورکومین + 5 میلی گرم پیپرین، در مجموع روزانه 1500 میلی گرم کورکومین و 15 میلی گرم پیپرین) و گروه کنترل روزانه سه کپسول دارونما (هر کپسول 500 میلی گرم مالتودکسترین) دریافت خواهند کرد. مکمل ها و دارونما همراه با گاواژ 6 صبح، گاواژ 12 ظهر و گاواژ 6 بعد از ظهر دریافت خواهد شد. در ابتدای مطالعه (قبل از شروع طرح) و انتهای هفته دوم (14 روز)، فاکتورهای التهابی (ESR و CRP)، شاخص های کبدی و کلیوی، CBC، قند و آلبومین خون، درجه حرارت بدن (تب)، میزان تنفس در دقیقه، آبریزش بینی، میزان و شدت سرفه و طول مدت بیماری ارزیابی می شوند. جهت تحلیل داده ها از نرم افزار SPSS ورژن 22 به منظور تحلیل داده ها با سطح معناداری P<0.05 استفاده خواهد شد. | | | |
| **کلیدواژه­ها** | | | |
| **سه تا پنج کلیدواژه به زبان انگلیسی و بر اساس MeSH**  Curcumin, Piperine, COVID-19, ICU | | | |
| **بودجه درخواستی از دانشگاه (ریال)** | 100000000 | **مدت زمان اجرا (ماه)** | 18 ماه |

**ب) اطلاعات طرح**

| **اهداف اختصاصی** | | | |
| --- | --- | --- | --- |
| 1- تعیین و مقایسه میانگین درجه حرارت بدن **گروههای مداخله و دارونما قبل و بعد از مداخله.**  2- تعیین و مقایسه میانگین شاخص های التهابیESR و CRP در **گروههای مداخله و دارونما قبل و بعد از مداخله.**  3- تعیین و مقایسه میانگین طول مدت بیماری بیماران در **گروههای مداخله و دارونما قبل و بعد از مداخله.**  4- تعیین و مقایسه میزان و شدت سرفه های بیماران در **گروههای** **مداخله و دارونما قبل و بعد از مداخله.**  5-تعیین و مقایسه میانگین ضریب تنفسی در **گروههای** مداخله و دارونما قبل و بعد از مداخله.  6- تعیین و مقایسه میانگین آنزیم های کبدی (ALT، AST، LDH) در **گروههای** **مداخله و دارونما قبل و بعد از مداخله.**  7- تعیین و مقایسه میانگین شاخص های کلیوی (BUN، Creatinine) در **گروههای** **مداخله و دارونما قبل و بعد از مداخله**  8- تعیین و مقایسه میانگین شاخص های مربوط به شمارش گلبول های خون (CBC) در **گروههای** **مداخله و دارونما قبل و بعد از مداخله**  9- تعیین و مقایسه میانگین آلبومین خون در **گروههای** **مداخله و دارونما قبل و بعد از مداخله**  **10-** تعیین و مقایسه میانگین قند خون در **گروههای** **مداخله و دارونما قبل و بعد از مداخله**  **11-** تعیین تأثیر مکمل کورکومین پیپرین بر نمرهNUTRIC ، نمره APACHE II و SOFA در بیماران مبتلا به ویروس کرونا بستری در بخش مراقبت های ویژه (ICU) و مقایسه آن با گروه کنترل | | | |
| **بیان مساله و ضرورت اجرای مطالعه** | | | |
| ویروس کرونا پاتوژن مهم انسانی و حیوانی است. در اواخر سال 2019 ، یک ویروس کرونای جدید به عنوان علت خوشه ای از موارد ذات الریه در ووهان چین شناسایی شد. ین بیماری به سرعت همه گیر شد، و علاوه بر چین در فوریه سال 2020 این بیماری در سراسر جهان مشاهده گردید. سازمان بهداشت جهانی نام این بیماری را COVID-19 را تعیین کرده است (1, 2). در واقع ویروس کرونای جدید که باعث مشکلات حاد ریوی میگردد همان SARS-CoV-2 (سندرم حاد تنفسی حاد نوع 2) می باشد که سازمان بهداشت جهانی آن را COVID-19 نامیده است (1). ویروس کرونا^[[5]](#footnote-5)^ شامل خانواده بزرگی از ویروس هاست که از بیماری سرماخوردگی تا بیماری های شدیدتر مانند سندرم تنفسی خاورمیانه (MERS-CoV)^[[6]](#footnote-6)^ و سندرم حاد تنفسی^[[7]](#footnote-7)^ (SARS-CoV) را شامل می شود (3, 4). ویروس کرونا جدید^[[8]](#footnote-8)^ (nCoV) نوعی ویروس جدید است که قبلاً در انسان مشخص نشده است. ویروس کرونا عامل ایجاد بیماری های تنفسی، دستگاه گوارش، کبدی و سیستم عصبی مرکزی در انسان، دام و حیوانات وحشی است. خفاش ها به عنوان حامل طبیعی انواع ویروس کرونا معرفی شده اند (5, 6). علائم شایع عفونت در این بیماری، شامل علائم تنفسی، تب، سرفه، تنگی نفس و مشکلات تنفسی است. در موارد شدیدتر، عفونت می تواند باعث ذات الریه، سندرم حاد تنفسی شدید، نارسایی کلیه و حتی مرگ شود (5). یکی از مهمترین عوارض ویروس کرونا سندرم حاد تنفسی حاد (SARS) می باشد. SARS در اواخر سال 2002 از جنوب چین سرچشمه گرفته و مرگ و میر و عوارض بالایی دارد. طی یک دوره شش ماهه از اواخر سال 2002 ، این بیماری بیش از 8000 نفر را مبتلا کرده و نزدیک به 800 نفر را کشته است (5). این بیماری تهدیدی جدید برای سلامت انسان ها محسوب می شود و یک چالش برای توسعه و تجویز داروهای ضد ویروسی است. SARS توسط یک ویروس کرونای جدید مرتبط با SARS ایجاد می شود (بیماری همه گیر پنومونی آتیپیک) که توسط یک شبکه آزمایشگاهی جهانی تحت نظارت سازمان بهداشت جهانی (WHO) شناسایی شده است (5, 7). این ویروس ها معمولا به عنوان بیماری تنفسی و دستگاه گوارش در انسان و حیوانات اهلی شناخته می شوند. اگرچه اتیولوژی این بیماری هنوز به طور کامل شناخته شده نیست اما افزایش التهاب ریه ها یکی از عواملی است که بصورت منفی بر بیماری اثر می گذارد (8). لذا در بعضی موارد برای کاهش التهاب و کنترل بهتر بیماری از داروهای ضد التهابی استروییدی استفاده گردیده که مشخص شده این امر موجب کاهش توان سیستم ایمنی بدن و در نتیجه کاهش مقاومت در برابر بیماری و اثرات نامطلوب بسیاری برای فرد می گردد. با توجه به اپیدمی شدن ناگهانی این بیماری، تاکنون راهکار درمانی مناسبی برای آن معرفی نشده است.  یکی از مواردی که می تواند موجب کاهش التهاب و بهبود وضعیت این بیماران گردد گیاهان دارویی است که مطالعات مختلف نشان داده اند که این گیاهان اثرات ضدالتهابی، ضد استرس اکسیداتیو، ضد باکتریایی و ضد عفونت دارند، در عین حال معمولا عارضه خاصی ندارند و میتوانند بعنوان طب مکمل برای بهبود وضعیت بیماری مورد استفاده قرار گیرد (9-14). از جمله این گیاهان کورکومین است. کورکومین دارای فعالیت های آنتی اکسیدانی، ضد التهابی، تعدیل کننده سیستم ایمنی، اثرات مطلوب روی مقاومت به انسولین، فشار خون، چربی خون، آنزیم های کبدی و بسیاری دیگر از شاخص های مرتبط با سلامتی انسان می باشد و می تواند یک ماده طبیعی بالقوه برای پیشگیری و یا درمان برخی از بیماری ها (15-20) باشد. همچنین یافته های مطالعات قبل بیانگر اثرات ضد میکروبی، ضد قارچی، ضد ویروس و ضد پنومونی بوده است (21-26). هم چنین مطالعات پره کلینیکال اخیر نشان داده اند که کورکومین می تواند بعنوان یک ماده گیاهی جدید اثرات مطلوبی بر آنفولانزا داشته باشد بطوریکه باعث کاهش تولید مثل ویروس، کاهش التهاب ریه ها و پنومونی میگردد (23, 27-29). همچنین اثرات مطلوب کورکومین بر روی بیماری های ریوی به خصوص آسیب و فیبروز ریه ها و کاهش التهاب ریه ها که در اثر بیماری های مختلف و همچنین پرتودرمانی، داروهای شیمی درمانی و سموم بوجود می آید را نشان داده اند (27, 30-34).  نتایج مطالعه مرور سیستماتیک اخیر نشاندهنده اثرات ضد میکروبی و اثرات مطلوب کورکومین بر شاخص های استرس اکسیداتیو، التهاب و سیستم ایمنی در سپسیس بر اساس مطالعات سلولی مولکولی و حیوانی می باشد (35). مشخص شده است که کورکومین توانایی مهار تکثیر سلولهای التهابی، تهاجم و رگ زایی را از طریق اهداف مولکولی متعدد و مکانیسم های عملکردی را داراست. کورکومین بی خطر و غیر سمی است و از طریق تنظیم فاکتورهای رونویسی التهابی، سیتوکین ها، وضعیت ردوکس، پروتئین کینازها و آنزیم هایی که همگی التهاب را تقویت می کنند، اثرات ضد التهابی آن را القا می کند. علاوه بر این، کورکومین از طریق مسیرهای میتوکندریایی و گیرنده و همچنین فعال شدن آبشارهای کاسپاز باعث آپوپتوز می شود (36). مطالعات بسیار نشان داده اند خواص ضد التهابی کورکومین از طریق افزایش آپوپتوز، غیر فعال کردن nuclear factor-kappa B (NF-kB) و کاهش بیان ژن آن، همچنین غیر فعال کردن سیتوکین های فراوانی مانند inflammatory cytokines such as tumor necrosis factor-alpha (TNF-a), interleukins (IL-1, -1b, -6, and -8), and cyclooxygenase-2 (COX-2) اعمال می شود (37).  در مطالعه حاضر مکمل غذایی تحت عنوان کورکومین (3 تا 5 درصد زردچوبه) می باشد که شامل دو جزو اصلی یعنی دمتوکسی کورکومین و بیس دمتوکسی کورکومین می باشد که در مجموع به آن ها کورکومینویید نیز گفته می شود (38-41). نکته حائز اهمیت درباره کورکومین اینست که کورکومین علیرغم پتانسیل درمانی بالا، به علت حلالیت آبی ضعیفی که دارد و همچنین زیست دسترسی پایین و تخریب سریع ان برای استفاده در اهداف پزشکی دارای محدودیت می باشد (42-44). بعبارت دیگر، مطالعات متعددی نشان داده اند که کورکومین حتی در دوزهای بالای 8 گرم در روز بی خطر است. با این حال، ناپایداری در pH فیزیولوژیکی، حلالیت کم در آب و متابولیسم سریع منجر به فراهمی زیستی خوراکی کم کورکومین می شود (42-45) با این حال، برای بهبود پارامترهای مرتبط با بالینی آن، فرمولاسیون کورکومین همراه با پیپرین به عنوان یک جایگزین جدید برای روش های درمانی برتر خود معرفی شده است. پیپرین از طریق افزایش زیست فراهمی کورکومین و کاهش کونژوگاسیون کبدی آن با گلوکورونیک اسید و در نتیجه کاهش دفع ادراری آن این محدودیت ها را برطرف می سازد (46). در واقع یکی از محبوبترین و بهترین ترکیبات کورکومین که در سال های اخیر درباره آن مطالعات زیادی منتشر شده است ترکیب آن با پیپرین می باشد (47-50). مطالعات قبلی نشان داده اند که این ترکیب کاملا ایمن و با جذب بالا و زیست دسترسی بالا نسبت به کورکومین تنها، دارای کارایی بسیار بالاتری برای کاهش التهاب در بدن و سایر خواص کورکومین می باشد. در این زمینه (کاربرد بالا و ایمن بودن ترکیب کورکومین پیپرین) حتی مقالات مروری نیز به چاپ رسیده اند (51-54). همچنین به ازای هر 500 میلیگرم کورکومین فقط 5 میلی گرم پیپرین بکار می رود. یعنی مقدار پیپرین بسیار ناچیز است و در واقع این مقدار بکار می رود تا در روده با آنزیم گلوکورینیداز^[[9]](#footnote-9)^ باند شود و اجازه جذب بهتر به کورکومین داده شود (55). لذا اعتقاد بر آن است که این مقدار ناچیز پیپرین جذب سیستمیک ندارد. اگر هم مقداری از آن جذب شود مطالعات قبلی بیانگر اثرات ضد التهابی و مفید پیپرین (بصورت مکمل با دوزهای بالاتر) هستند (56-58) که باز هم این موضوع باعث بهبود وضعیت بیماران می گردد.  با توجه به عدم وجود راهکار درمانی قطعی برای درمان کرونا و عدم وجود واکسن و .... و با در نظر گرفتن خواص متعدد کورکومین که امروزه در اکثر بیماری ها بعنوان یک مکمل ایمن و پرکاربرد کارایی دارد و با توجه به اثرات مطلوب کورکومین بر روی بیماری های ویروسی (59, 60)، پنومومنی (61-65)، سایر عفونت های باکتریایی (24, 26)، هم چنین اثرات بسیار خوب کورکومین بر روی آسیب ریه ها (66-70) و کاهش التهاب آن ها (71, 72)، اثرات مطلوب کورکومین بر روی سیستم ایمنی (73, 74)، خواص ضد التهابی و آنتی اکسیدانی (75-77) آن بنظر می رسد این مکمل غذایی میتواند بعنوان یک مکمل ایمن (در مطالعات قبلی ایمن بودن این مکمل به اثبات رسیده است)، طبیعی و ارزان قیمت اثرات بسیار مطلوبی بر وضعیت سلامتی این بیماران داشته باشد. لازم به ذکر است که کورکومین و آنالوگ های آن برای درمان سرطان نیز پیشنهاد شده اند (78-81).  با توجه به عدم درمان مناسب و قطعی برای انواع بیماری های ویروسی مانند آنفولانزا و کرونا و عوارض و پیامدهای بیشمار این بیماری ها که منجر به تحمیل خسارت های بیشمار به سیستم بهداشتی کشورها می گردد بنظر می رسد انجام مداخلات جهت یافتن راهکاری مناسب و مقرون به صرفه اهمیت فوق العاده ای دارد.  با عنایت به اثرات بسیار مناسب کورکومین بر جنبه های مختلف سلامتی انسان و اثرات مطلوب آن بر بیماری های عفونی مانند آنفولانزا و پنومونی در مطالعات پایه همچنین اثرات مطلوب کورکومین بر شاخص های استرس اکسیداتیو، التهابی و سیستم ایمنی و اینکه مطالعات اخیر بیانگر فواید احتمالی کورکومین در بیماران کرونایی می باشد (82) و با توجه به اینکه در مطالعات قبلی کورکومین اثرات مطلوبی در بیماران آی سی یو داشته است (83-85) نظر می رسد استفاده از مکمل کورکومین پیپرین بعنوان یک مکمل غذایی گیاهی، طبیعی، ارزان قیمت، در دسترس و بدون عارضه در بیماران مبتلا به ویروس کرونا می تواند باعث بهبود وضعیت این بیماران گردد. لذا هدف از مطالعه حاضر بررسی اثر مکمل یاری با کورکومین پیپرین (سه کپسول هر کپسول حاوی 500 میلی گرمی کورکومین + 5 میلی گرم پیپرین در روز برای دو هفته؛ در مجموع 14 روز هر روز 1500 میلی گرم کورکومین + 15 میلی گرم پیپرین) بر روی بیماران مبتلا به ویروس کرونا بستری در بخش مراقبت های ویژه (ICU) طی یک مطالعه کارآزمایی بالینی دو سو کور می باشد. امید است نتایج این مطالعه بتواند باعث بهبود وضعیت سلامتی بیماران مبتلا به ویروس کرونا بستری در ICU گردد. | | | |
| **روش اجرا** | | | |
| ***نوع مطالعه و روش پژوهش***  کارآزمایی بالینی تصادفی شده دوسویه کور و کنترل شده با دارونما  ***جامعه آماری پژوهش***  مطالعه­ی کارآزمایی بالینی تصادفی سازی شده دوسوکور و کنترل شده با دارونما در سال 1399 بر روی 50 بیمار بالغ 20 تا 65 سال مبتلا به ویروس کرونا در بیمارستان های وابسته به دانشگاه علوم پزشکی اصفهان (در صورتیکه بیمارستان الزهرا (س) بیمارستان کرونا باشد در این بیمارستان اجرا می شود) انجام خواهد گرفت.  ***معیارهای ورود به مطالعه***   - تمایل به شرکت در مطالعه - سن 20-65 سال - تشخیص ابتلا به Covid-19 براساس یافته های بالینی و یافته های حاصل از PCR - دستگاه گوارش با عملکرد نرمال و دارای معیارهای تغذیه روده ای   **معیار های عدم ورود به مطالعه**   - *سن کمتر از 20 و بیشتر از 65 سال* - *حساسیت به فرآورده های گیاهی مانند زردچوبه و فلفل* - عدم امکان تغذیه روده ای در 48 ساعت اول پذیرش - بیمارانی که کمتر از 48 ساعت در بخش مراقبت های ویژه بستری باشند. - بیمارانی که پیش بینی می شود ظرف 12 ساعت از پذیرش در بخش مراقبت های ویژه فوت کنند. - بیمارانی که در روز اول اندیکاسیون تغذیه روده ای را ندارند و براساس تشخیص بخش مراقبت های ویژه تایید و پیش بینی می شود که در آینده نیز قادر به دریافت تغذیه روده ای نمی باشند. (تهوع، استفراغ مقاوم، ایلئوس، انسداد روده، اسهال کنترل نشده (> 500 میلی لیتر در روز)، فیستول با خروجی بالا (> 500 میلی لیتر در روز)، عدم دسترسی به روده، احیاء ناقص و بی ثباتی همودینامیک - بیمارانی با نمایه توده بدنی BMI<18.5kg/m^2^ که در بخش مراقبت های ویژه پذیرش می شوند. - بیمارانی که تحت حمایت تغذیه ای به روش تغذیه وریدی کامل قرار می گیرند. - بیمارانی که سابقه بیماری زمینه ای از قبیل اختلالات مادرزادی و ایمنی، نارسایی کلیوی و کبدی و پانکراتیت دارند. - مصرف داروهای ضد انعقاد خون مانند هپارین، وارفارین، آسپیرین و غیره - بارداری و شیردهی - شوک سپتیک یا سپسیس شدید - عدم رضایت بیمار یا ولی قانونی وی   **معیارهای خروج از مطالعه**   - *عدم تمایل به ادامه همکاری* - *مشاهده هرگونه عوارض جانبی، ایجاد هر یک از شرایط عدم ورود در حین مطالعه* - دریافت خون مکرر در بیماران. - افرادی که تغذیه انترال را خوب تحمل نکنند. - ایجاد پروسه های عفونی، DIC(انعقاد منتشر داخل عروقی) و هر گونه تعاملات التهابی که در روند مداخله تداخل ایجاد نماید. - مصرف کمتر از 90% مکمل کورکومین-پیپرین - عوارض جانبی که میتوانند پس از مصرف مکمل موجب توقف طرح یا خروج آزمودنی از مطالعه شوند:   ایجاد هر گونه مشکل گوارشی نا خواسته شامل دل درد، دل پیچه، تهوع، استفراغ، اسهال یا غیره  ایجاد هر گونه حساسیت اعم از پوستی، تنفسی یا غیره  ایجاد هر گونه عارضه نامطلوبی که تا قبل از مصرف مکمل وجود نداشته است  ***روش گردآوری داده و نمونه***  پس از انتخاب شرکت کنندگان بر اساس معیارهای ورود و کسب رضایت نامه از بیماران یا همراه آن ها (پیوست شماره 1)، شرکت کنندگان به صورت تصادفی به دو گروه، مداخله و دارونما تقسیم و به مدت 2 هفته (14 روز) مورد مطالعه قرار خواهند گرفت. برای این منظور 50 بیمار بستری در بخش ICU که از قبل ابتلای آنها به COVID19 توسط PCR تشخیص داده شده است با استفاده از جدول اعداد تصادفی به طور تصادفی به دو گروه تقسیم خواهند شد (25 نفر گروه مداخله و 25 نفر گروه کنترل).  گروه 1) بیمارانی که به مدت 14 روز 3 کپسول 500 میلی گرمی در روز کورکومین-پیپرین دریافت می کنند (در مجموع روزی 1500 میلی گرم کورکومین و روزی 15 میلی گرم پیپرین در روز) (25 نفر)  گروه 2) بیمارانی که به مدت 14 روز 3 کپسول دارونما هر کپسول حاوی 500 میلی گرم مالتودکسترین در روز دریافت می کنند (در مجموع 1500 میلی گرم مالتودکسترین) (25 نفر)  در گروهها یک کپسول بعد از گاواژ ساعت 6 صبح،یک کپسول بعد از گاواژ 12 ظهر و یک کپسول بعد از گاواژ ساعت 6 عصر انجام به بیمار داده میشود.( همراه با گاواژ)  جهت رعایت اصول اخلاقی هیچ مداخله ای در زمینه دارو درمانی بیماران انجام نمی شود. گاواژ همه بیماران بصورت یکسان طبق آخرین دستورالعمل تغذیه بیماران کرونا طبق نظر متخصص آی سی یو بیهوشی و طبق روال بیمارستان برای همه بیماران بستری در آی سی یو تنظیم میشود و توسط واحد آشپزخانه و کارشناسان تغذیه بیمارستان الزهرا (س) تهیه می گردد. جهت دوسوکور اجرا کردن این تحقیق، قبل از شروع مطالعه مجموع کپسول های مربوطه توسط فردی غیر از پژوهشگر به صورت A و B کدگذاری می شوند تا عدم اطلاع محقق از نوع کپسول های دریافتی توسط هر دو گروه رعایت شود. مکمل ها و دارونما بعد از گاواژ ساعت 6 صبح و 6 عصر به بیماران داده می شود.  کپسول های کورکومین/پیپرین و پلاسبو از شرکت **Sami labs Ltd., India** خریداری می گردد ایمن بودن این محصول در مطالعات قبلی نشان داده شده است (47-50). کپسول ها از نظر نوع، رنگ، شکل و اندازه کاملا یکسان می باشند.  ***فرمولای تغذیه ای مورد استفاده***  روش تغذیه ای تمام بیماران در هر دو گروه تغذیه انترال می باشد و در همه نمونه ها در 24-48 ساعت اول بستری آغاز می شود. هر دو گروه از فرمولای استاندارد بیمارستان طبق روال عادی استفاده می کنند.  ***حجم نمونه***  تعداد نمونه با در نظر گرفتن خطای نوع اول α=0.05 و خطای نوع دوم β=0.20 با توان آزمون 80% و اندازه ی اثر (effect size) استاندارد شده برابر با Δ =12 از فرمول زیر بر اساس شاخص CRP محاسبه می شود (86):  n= 2[(Z_1-α/2_ + Z1-β)^2^ × S^2^] / Δ2=2[(1.96+0.84)^2^ × (4)^2^] / (3.5)^2^  که حجم نمونه مورد نیاز در هر گروه 20 نفر تعیین گردید که با در نظر گرفتن ریزش افراد، حجم نمونه عملاً 25 نفر در هر گروه و در مجموع 50 نفر در نظر خواهیم گرفت.  ***جمع آوری داده ها***  تمامی شاخص های زیر طبق پرسش نامه ضمیمه توسط پرستار بخش (برای هر بیمار یک پرستار مشخص برای قبل و بعد بیماری) و زیر نظر پزشک متخصص آی سی یو بیهوشی در نظر گرفته می شود.  **متغیر های مورد ارزیابی**  به طور کلی متغیرهای مورد ارزیابی در این مطالعه به متغیرهای دموگرافیک (پیوست شماره 2)، ارزیابی شدت بیماری، فاکتورهای التهابی، تغذیه ای و فاکتورهای ارزیابی شدت بیماری (پیوست شماره 3) تقسیم می شوند.  ***مشخصات دموگرافیک***  متغیرهای دموگرافیک (شامل سن، جنس، وضعیت تاهل، استعمال دخانیات، سابقه ی بیماری و تاریخچه پزشکی، سطح تحصیلات، شغل، مصرف مکمل ها و داروها) با تکمیل پرسشنامه اطلاعات عمومی از پرونده بیماران جمع آوری می شود.  ***خونگیری***  در روزهای صفر (قبل از شروع مداخله) و روز 14 (پایان مداخله)، به میزان 10 میلی لیتر خون بعد از آخرین وعده گاواژ شبانه از بیمار گرفته می شود و در لوله ی پلاستیکی جمع آوری می شود برای جداکردن سرم نمونه ها، آن ها را در سانتریفیوژ با 3600 دور به مدت 3-4 دقیقه گذاشته و در میکروتیوب های حاوی 0.5 میلی لیتر ریخته می شود. نمونه ها در فریزر -20^°C^  نگهداری می شود. برای کاهش خطا در اندازه گیری ها، تمام نمونه گیری ها در ساعت 8-10 صبح و در وضعیت ناشتا انجام می شود.  ***اندازه گیری های بیوشیمیایی***  CRP، ESR سرم با استفاده از روش آنزیمی مشخص می شود.  میزان آنزیم های کبدی (ALT,AST، LDH) سرم با استفاده از روش فتومتریک آنزیماتیک (IFCC) کیت کلریمتریک تعیین می گردد.  ***آنالیز آماری***  در مطالعه حاضر حجم متغیرهای کمی به صورت میانگین (انحراف معیار) و متغیرهای کیفی به صورت تعداد (درصد) گزارش خواهند شد. ارزیابی نرمال بودن توزیع متغیرهای کمی با استفاده از شاخص sksewnes و نمودار Q-Q plot انجام خواهد گرفت. تحلیل های درون گروهی با استفاده از آزمون تی زوجی و تحلیل های بین گروهی با استفاده از آزمون تی مستقل و ANCOVA انجام خواهد گرفت. توزیع متغیرهای کیفی با استفاده از آزمون کای دو بین دو گروه مقایسه خواهد شد. جهت تحلیل داده ها از نرم افزار SPSS ورژن 22 به منظور تحلیل داده ها با سطح معناداری P<0.05 استفاده خواهد شد. | | | |
| **متغیرهای مطالعه** | | | |
| **نام متغیر** | **نقش** (مستقل یا وابسته) | **نوع** (کیفی اسمی، کیفی رتبه­ای، کمی) | **واحد اندازه گیری** |
| مکمل کورکومین/پیپرین | مستقل | کیفی اسمی | عدد |
| درجه حرارت بدن | وابسته | کمی پیوسته | عدد |
| طول مدت بستری | وابسته | کمی گسسته | عدد |
| CRP | وابسته | کمی پیوسته | mg/dl |
| ESR | وابسته | کمی پیوسته | mg/dl |
| ALT | وابسته | کمی پیوسته | mg/dl |
| AST | وابسته | کمی پیوسته | mg/dl |
| LDH | وابسته | کمی پیوسته | mg/dl |
| BUN | وابسته | کمی پیوسته | mg/dl |
| Creatinine | وابسته | کمی پیوسته | mg/dl |
| CBC | وابسته | کمی پیوسته | عدد |
| آلبومین خون | وابسته | کمی پیوسته | mg/dl |
| قند خون | وابسته | کمی پیوسته | mg/dl |
| شدت بیماری | وابسته | کیفی گسسته | عدد |
| بیماری زمینه ای | مخدوشگر | کیفی اسمی | نوع بیماری |
| سن | زمینه ای | کمی گسسته | سال |
| جنس | زمینه ای | کیفی اسمی | مرد/زن |
| **دستاوردهای مورد انتظار** | | | |
| با توجه به استفاده از کورکومین/پیپرین برای اولین بار در درمان ویروس کرونا بستری در آی سی یو ممکن است این مکمل غذایی بدون عارضه و طبیعی در آینده نزدیک برای بهبود وضعیت بیماران مبتلا به این ویروس معرفی گردد. | | | |
| **محدودیت­ها** | | | |
| عدم همکاری برخی از مراجعین، که برای ترغیب آنها به شرکت در مطالعه درباره ی تاثیرات مکمل کورکومین/پیپرین بر روی بیماری توضیح داده خواهد شد، همچنین به آنها ذکر می شود که هزینه ی تمامی آزمایشات در طی مداخله رایگان میباشد.  عدم تبعیت از مصرف مکمل مورد مطالعه که با پیگیری پرستاران و یادآوری مصرف مکمل مورد مطالعه این مشکل نیز حل خواهد شد. | | | |
| **منافع تجاری احتمالی** | | | |
| **اگر پیش بینی می­کنید این مطالعه دستاوردهایی با قابلیت تجاری سازی داشته باشد، آنها را تشریح کنید.**  ندارد. | | | |
| **منابع** | | | |
| **حداکثر 50 منبع و طبق الگوی ونکوور**  1. McIntosh K. Coronavirus disease 2019 (COVID-19).  2. Zhu N, Zhang D, Wang W, Li X, Yang B, Song J, et al. A novel coronavirus from patients with pneumonia in China, 2019. New England Journal of Medicine. 2020.  3. Barrea L, Altieri B, Muscogiuri G, Laudisio D, Annunziata G, Colao A, et al. Impact of nutritional status on gastroenteropancreatic neuroendocrine tumors (GEP-NET) aggressiveness. Nutrients. 2018;10(12).  4. Lorusso A, Calistri P, Petrini A, Savini G, Decaro N. Novel coronavirus (SARS-CoV-2) epidemic: a veterinary perspective. Veterinaria Italiana. 2020.  5. Groneberg DA, Hilgenfeld R, Zabel P. Molecular mechanisms of severe acute respiratory syndrome (SARS). Respiratory Research. 2005;6(1):8.  6. Munster VJ, Koopmans M, van Doremalen N, van Riel D, de Wit E. A novel coronavirus emerging in China—key questions for impact assessment. New England Journal of Medicine. 2020;382(8):692-4.  7. Xu X-W, Wu X-X, Jiang X-G, Xu K-J, Ying L-J, Ma C-L, et al. Clinical findings in a group of patients infected with the 2019 novel coronavirus (SARS-Cov-2) outside of Wuhan, China: retrospective case series. bmj. 2020;368.  8. Clay C, Donart N, Fomukong N, Knight JB, Lei W, Price L, et al. Primary severe acute respiratory syndrome coronavirus infection limits replication but not lung inflammation upon homologous rechallenge. Journal of virology. 2012;86(8):4234-44.  9. Tilburt JC, Kaptchuk TJ. Herbal medicine research and global health: an ethical analysis. Bulletin of the World Health Organization. 2008;86:594-9.  10. Bent S. Herbal medicine in the United States: review of efficacy, safety, and regulation. Journal of general internal medicine. 2008;23(6):854-9.  11. Pavithra P, Janani V, Charumathi K, Indumathy R, Potala S, Verma RS. Antibacterial activity of plants used in Indian herbal medicine. International Journal of Green Pharmacy (IJGP). 2010;4(1).  12. Shahidi Bonjar G, Aghighi S, Karimi Nik A. Antibacterial and antifungal survey in plants used in indigenous herbal-medicine of south east regions of Iran. Journal of Biological Sciences. 2004;4(3):405-12.  13. He D-Y, Dai S-M. Anti-inflammatory and immunomodulatory effects of Paeonia lactiflora Pall., a traditional Chinese herbal medicine. Frontiers in pharmacology. 2011;2:10.  14. Ke F, Yadav PK, Ju LZ. Herbal medicine in the treatment of ulcerative colitis. Saudi journal of gastroenterology: official journal of the Saudi Gastroenterology Association. 2012;18(1):3.  15. Leclercq IA, Farrell GC, Sempoux C, dela Peña A, Horsmans Y. Curcumin inhibits NF-κB activation and reduces the severity of experimental steatohepatitis in mice. Journal of hepatology. 2004;41(6):926-34.  16. Wu S-J, Lin Y-H, Chu C-C, Tsai Y-H, Chao JC-J. Curcumin or saikosaponin a improves hepatic antioxidant capacity and protects against CCl4-induced liver injury in rats. Journal of medicinal food. 2008;11(2):224-9.  17. Kuo J-J, Chang H-H, Tsai T-H, Lee T-Y. Positive effect of curcumin on inflammation and mitochondrial dysfunction in obese mice with liver steatosis. International journal of molecular medicine. 2012;30(3):673-9.  18. Thota RN, Acharya SH, Garg ML. Curcumin and/or omega-3 polyunsaturated fatty acids supplementation reduces insulin resistance and blood lipids in individuals with high risk of type 2 diabetes: a randomised controlled trial. Lipids in health and disease. 2019;18(1):31.  19. Poolsup N, Suksomboon N, Kurnianta PDM, Deawjaroen K. Effects of curcumin on glycemic control and lipid profile in prediabetes and type 2 diabetes mellitus: A systematic review and meta-analysis. PloS one. 2019;14(4):e0215840.  20. Zhang D, Huang C, Yang C, Liu RJ, Wang J, Niu J, et al. Antifibrotic effects of curcumin are associated with overexpression of cathepsins K and L in bleomycin treated mice and human fibroblasts. Respiratory research. 2011;12(1):154.  21. Zhang B, Swamy S, Balijepalli S, Panicker S, Mooliyil J, Sherman MA, et al. Direct pulmonary delivery of solubilized curcumin reduces severity of lethal pneumonia. FASEB journal : official publication of the Federation of American Societies for Experimental Biology. 2019;33(12):13294-309.  22. Wang J, Zhou X, Li W, Deng X, Deng Y, Niu X. Curcumin protects mice from Staphylococcus aureus pneumonia by interfering with the self-assembly process of alpha-hemolysin. Scientific reports. 2016;6:28254.  23. Dai J, Gu L, Su Y, Wang Q, Zhao Y, Chen X, et al. Inhibition of curcumin on influenza A virus infection and influenzal pneumonia via oxidative stress, TLR2/4, p38/JNK MAPK and NF-kappaB pathways. International immunopharmacology. 2018;54:177-87.  24. Mun S-H, Joung D-K, Kim Y-S, Kang O-H, Kim S-B, Seo Y-S, et al. Synergistic antibacterial effect of curcumin against methicillin-resistant Staphylococcus aureus. Phytomedicine. 2013;20(8-9):714-8.  25. Rai D, Singh JK, Roy N, Panda D. Curcumin inhibits FtsZ assembly: an attractive mechanism for its antibacterial activity. Biochemical Journal. 2008;410(1):147-55.  26. Zorofchian Moghadamtousi S, Abdul Kadir H, Hassandarvish P, Tajik H, Abubakar S, Zandi K. A review on antibacterial, antiviral, and antifungal activity of curcumin. BioMed research international. 2014;2014.  27. Xu Y, Liu L. Curcumin alleviates macrophage activation and lung inflammation induced by influenza virus infection through inhibiting the NF-kappaB signaling pathway. Influenza and other respiratory viruses. 2017;11(5):457-63.  28. Umar S, Shah MA, Munir MT, Yaqoob M, Fiaz M, Anjum S, et al. Synergistic effects of thymoquinone and curcumin on immune response and anti-viral activity against avian influenza virus (H9N2) in turkeys. Poultry science. 2016;95(7):1513-20.  29. Han S, Xu J, Guo X, Huang M. Curcumin ameliorates severe influenza pneumonia via attenuating lung injury and regulating macrophage cytokines production. Clinical and experimental pharmacology & physiology. 2018;45(1):84-93.  30. Cheng K, Yang A, Hu X, Zhu D, Liu K. Curcumin Attenuates Pulmonary Inflammation in Lipopolysaccharide Induced Acute Lung Injury in Neonatal Rat Model by Activating Peroxisome Proliferator-Activated Receptor gamma (PPARgamma) Pathway. Medical science monitor : international medical journal of experimental and clinical research. 2018;24:1178-84.  31. Venkatesan N, Punithavathi D, Babu M. Protection from acute and chronic lung diseases by curcumin. Advances in experimental medicine and biology. 2007;595:379-405.  32. Xiao Z, Xu F, Zhu X, Bai B, Guo L, Liang G, et al. Inhibition Of JNK Phosphorylation By Curcumin Analog C66 Protects LPS-Induced Acute Lung Injury. Drug design, development and therapy. 2019;13:4161-71.  33. Chai YS, Chen YQ, Lin SH, Xie K, Wang CJ, Yang YZ, et al. Curcumin regulates the differentiation of naive CD4+T cells and activates IL-10 immune modulation against acute lung injury in mice. Biomedicine & pharmacotherapy = Biomedecine & pharmacotherapie. 2020;125:109946.  34. Almatroodi SA, Alrumaihi F, Alsahli MA, Alhommrani MF, Khan A, Rahmani AH. Curcumin, an Active Constituent of Turmeric Spice: Implication in the Prevention of Lung Injury Induced by Benzo(a) Pyrene (BaP) in Rats. Molecules (Basel, Switzerland). 2020;25(3).  35. Karimi A, Ghodsi R, Kooshki F, Karimi M, Asghariazar V, Tarighat-Esfanjani A. Therapeutic effects of curcumin on sepsis and mechanisms of action: A systematic review of preclinical studies. Phytotherapy research : PTR. 2019;33(11):2798-820.  36. Shehzad A, Rehman G, Lee YS. Curcumin in inflammatory diseases. Biofactors. 2013;39(1):69-77.  37. Shehzad A, Wahid F, Lee YS. Curcumin in cancer chemoprevention: molecular targets, pharmacokinetics, bioavailability, and clinical trials. Archiv der Pharmazie. 2010;343(9):489-99.  38. Chainoglou E, Hadjipavlou-Litina D. Curcumin analogues and derivatives with anti-proliferative and anti-inflammatory activity: Structural characteristics and molecular targets. Expert Opinion on Drug Discovery. 2019;14(8):821-42.  39. Mesa MD, Aguilera CM, Ramírez-Tortosa CL, Ramírez-Tortosa MC, Quiles JL, Baró L, et al. Oral administration of a turmeric extract inhibits erythrocyte and liver microsome membrane oxidation in rabbits fed with an atherogenic diet. Nutrition. 2003;19(9):800-4.  40. Tamvakopoulos C, Dimas K, Sofianos ZD, Hatziantoniou S, Han Z, Liu Z-L, et al. Metabolism and anticancer activity of the curcumin analogue, dimethoxycurcumin. Clinical Cancer Research. 2007;13(4):1269-77.  41. Teymouri M, Barati N, Pirro M, Sahebkar A. Biological and pharmacological evaluation of dimethoxycurcumin: A metabolically stable curcumin analogue with a promising therapeutic potential. Journal of cellular physiology. 2018;233(1):124-40.  42. Gera M, Sharma N, Ghosh M, Huynh DL, Lee SJ, Min T, et al. Nanoformulations of curcumin: an emerging paradigm for improved remedial application. Oncotarget. 2017;8(39):66680-98.  43. Anand P, Kunnumakkara AB, Newman RA, Aggarwal BB. Bioavailability of curcumin: problems and promises. Molecular pharmaceutics. 2007;4(6):807-18.  44. Siviero A, Gallo E, Maggini V, Gori L, Mugelli A, Firenzuoli F, et al. Curcumin, a golden spice with a low bioavailability. Journal of Herbal Medicine. 2015;5(2):57-70.  45. Mirzaei H, Shakeri A, Rashidi B, Jalili A, Banikazemi Z, Sahebkar A. Phytosomal curcumin: A review of pharmacokinetic, experimental and clinical studies. Biomedicine & pharmacotherapy = Biomedecine & pharmacotherapie. 2017;85:102-12.  46. Cicero AFG, Sahebkar A, Fogacci F, Bove M, Giovannini M, Borghi C. Effects of phytosomal curcumin on anthropometric parameters, insulin resistance, cortisolemia and non-alcoholic fatty liver disease indices: a double-blind, placebo-controlled clinical trial. European journal of nutrition. 2019.  47. Panahi Y, Badeli R, Karami GR, Sahebkar A. Investigation of the efficacy of adjunctive therapy with bioavailability‐boosted curcuminoids in major depressive disorder. Phytotherapy Research. 2015;29(1):17-21.  48. Esmaily H, Sahebkar A, Iranshahi M, Ganjali S, Mohammadi A, Ferns G, et al. An investigation of the effects of curcumin on anxiety and depression in obese individuals: A randomized controlled trial. Chinese journal of integrative medicine. 2015;21(5):332-8.  49. Rahimnia A-R, Panahi Y, Alishiri G, Sharafi M, Sahebkar A. Impact of supplementation with curcuminoids on systemic inflammation in patients with knee osteoarthritis: findings from a randomized double-blind placebo-controlled trial. Drug research. 2015;65(10):521-5.  50. Saberi-Karimian M, Keshvari M, Ghayour-Mobarhan M, Salehizadeh L, Rahmani S, Behnam B, et al. Effects of curcuminoids on inflammatory status in patients with non-alcoholic fatty liver disease: A randomized controlled trial. Complementary Therapies in Medicine. 2020;49:102322.  51. Li Q, Zhai W, Jiang Q, Huang R, Liu L, Dai J, et al. Curcumin–piperine mixtures in self-microemulsifying drug delivery system for ulcerative colitis therapy. International journal of pharmaceutics. 2015;490(1-2):22-31.  52. Kakarala M, Brenner DE, Korkaya H, Cheng C, Tazi K, Ginestier C, et al. Targeting breast stem cells with the cancer preventive compounds curcumin and piperine. Breast cancer research and treatment. 2010;122(3):777-85.  53. Shoba G, Joy D, Joseph T, Majeed M, Rajendran R, Srinivas P. Influence of piperine on the pharmacokinetics of curcumin in animals and human volunteers. Planta medica. 1998;64(04):353-6.  54. Moorthi C, Kathiresan K. Curcumin–Piperine/Curcumin–Quercetin/Curcumin–Silibinin dual drug-loaded nanoparticulate combination therapy: A novel approach to target and treat multidrug-resistant cancers. Journal of Medical Hypotheses and Ideas. 2013;7(1):15-20.  55. YAN W-l, HUANG Z-s, ZENG X-h, ZHAO Z-m, HUANG X-j, FANG C-f. Absorption Mechanism of Curcumin across Caco-2 Cell Model. Pharmacy Today. 2011(11):8.  56. Lu Y, Liu J, Li H, Gu L. Piperine ameliorates lipopolysaccharide-induced acute lung injury via modulating NF-κB signaling pathways. Inflammation. 2016;39(1):303-8.  57. Mujumdar AM, Dhuley JN, Deshmukh VK, Raman PH, Naik SR. Anti-inflammatory activity of piperine. Japanese Journal of Medical Science and Biology. 1990;43(3):95-100.  58. Bang JS, Choi HM, Sur B-J, Lim S-J, Kim JY, Yang H-I, et al. Anti-inflammatory and antiarthritic effects of piperine in human interleukin 1β-stimulated fibroblast-like synoviocytes and in rat arthritis models. Arthritis research & therapy. 2009;11(2):R49.  59. Dao TT, Nguyen PH, Won HK, Kim EH, Park J, Won BY, et al. Curcuminoids from Curcuma longa and their inhibitory activities on influenza A neuraminidases. Food chemistry. 2012;134(1):21-8.  60. Chen D-Y, Shien J-H, Tiley L, Chiou S-S, Wang S-Y, Chang T-J, et al. Curcumin inhibits influenza virus infection and haemagglutination activity. Food Chemistry. 2010;119(4):1346-51.  61. Wang J, Zhou X, Li W, Deng X, Deng Y, Niu X. Curcumin protects mice from Staphylococcus aureus pneumonia by interfering with the self-Assembly process of α-hemolysin. Scientific Reports. 2016;6.  62. Xu F, Diao R, Liu J, Kang Y, Wang X, Shi L. Curcumin attenuates staphylococcus aureus-induced acute lung injury. Clinical Respiratory Journal. 2015;9(1):87-97.  63. Ye Y, Li Y, Fang F. Upconversion nanoparticles conjugated with curcumin as a photosensitizer to inhibit methicillin-resistant Staphylococcus aureus in lung under near infrared light. International journal of nanomedicine. 2014;9:5157-65.  64. Zhang B, Swamy S, Balijepalli S, Panicker S, Mooliyil J, Sherman MA, et al. Direct pulmonary delivery of solubilized curcumin reduces severity of lethal pneumonia. FASEB journal : official publication of the Federation of American Societies for Experimental Biology. 2019;33(12):13294-309.  65. Dai J, Gu L, Su Y, Wang Q, Zhao Y, Chen X, et al. Inhibition of curcumin on influenza A virus infection and influenzal pneumonia via oxidative stress, TLR2/4, p38/JNK MAPK and NF-κB pathways. International Immunopharmacology. 2018;54:177-87.  66. Sun J, Guo W, Ben Y, Jiang J, Tan C, Xu Z, et al. Preventive effects of curcumin and dexamethasone on lung transplantation-associated lung injury in rats. Critical care medicine. 2008;36(4):1205-13.  67. Bansal S, Chhibber S. Curcumin alone and in combination with augmentin protects against pulmonary inflammation and acute lung injury generated during Klebsiella pneumoniae B5055-induced lung infection in BALB/c mice. Journal of medical microbiology. 2010;59(4):429-37.  68. Sun J, Yang D, Li S, Xu Z, Wang X, Bai C. Effects of curcumin or dexamethasone on lung ischaemia–reperfusion injury in rats. European Respiratory Journal. 2009;33(2):398-404.  69. Smith MR, Gangireddy SR, Narala VR, Hogaboam CM, Standiford TJ, Christensen PJ, et al. Curcumin inhibits fibrosis-related effects in IPF fibroblasts and in mice following bleomycin-induced lung injury. American journal of physiology-lung cellular and molecular physiology. 2010;298(5):L616-L25.  70. Xiao X, Yang M, Sun D, Sun S. Curcumin protects against sepsis-induced acute lung injury in rats. Journal of Surgical Research. 2012;176(1):e31-e9.  71. Cho YJ, Yi CO, Jeon BT, Jeong YY, Kang GM, Lee JE, et al. Curcumin attenuates radiation-induced inflammation and fibrosis in rat lungs. The Korean Journal of Physiology & Pharmacology. 2013;17(4):267-74.  72. Moghaddam S, Barta P, Mirabolfathinejad S, Ammar-Aouchiche Z, Garza NT, Vo T, et al. Curcumin inhibits COPD-like airway inflammation and lung cancer progression in mice. Carcinogenesis. 2009;30(11):1949-56.  73. Gautam SC, Gao X, Dulchavsky S. Immunomodulation by curcumin. The Molecular Targets and Therapeutic Uses of Curcumin in Health and Disease: Springer; 2007. p. 321-41.  74. Jagetia GC, Aggarwal BB. “Spicing up” of the immune system by curcumin. Journal of clinical immunology. 2007;27(1):19-35.  75. Jayaprakasha GK, Rao LJ, Sakariah KK. Antioxidant activities of curcumin, demethoxycurcumin and bisdemethoxycurcumin. Food chemistry. 2006;98(4):720-4.  76. Ak T, Gülçin İ. Antioxidant and radical scavenging properties of curcumin. Chemico-biological interactions. 2008;174(1):27-37.  77. Menon VP, Sudheer AR. Antioxidant and anti-inflammatory properties of curcumin. The molecular targets and therapeutic uses of curcumin in health and disease: Springer; 2007. p. 105-25.  78. Dhillon N, Aggarwal BB, Newman RA, Wolff RA, Kunnumakkara AB, Abbruzzese JL, et al. Phase II trial of curcumin in patients with advanced pancreatic cancer. Clinical Cancer Research. 2008;14(14):4491-9.  79. Johnson JJ, Mukhtar H. Curcumin for chemoprevention of colon cancer. Cancer letters. 2007;255(2):170-81.  80. Anand P, Sundaram C, Jhurani S, Kunnumakkara AB, Aggarwal BB. Curcumin and cancer: an “old-age” disease with an “age-old” solution. Cancer letters. 2008;267(1):133-64.  81. Bisht S, Feldmann G, Soni S, Ravi R, Karikar C, Maitra A, et al. Polymeric nanoparticle-encapsulated curcumin (" nanocurcumin"): a novel strategy for human cancer therapy. Journal of nanobiotechnology. 2007;5(1):3.  82. Babaei F, Nassiri‐Asl M, Hosseinzadeh H. Curcumin (a constituent of turmeric): New treatment option against COVID‐19. Food Science & Nutrition. 2020.  83. Shadnoush M, Zahedi H, Norouzy A, Sahebkar A, Sadeghi O, Najafi A, et al. Effects of supplementation with curcuminoids on serum adipokines in critically ill patients: a randomized double‐blind placebo‐controlled trial. Phytotherapy Research. 2020.  84. Karimi A, Mahmoodpoor A, Kooshki F, Niazkar HR, Tarighat-Esfanjani A. Effects of Nanocurcumin on Inflammatory Factors and Clinical Outcomes in Critically Ill Patients with sepsis: A pilot randomized clinical trial. European Journal of Integrative Medicine. 2020:101122.  85. Zahedi H, Hosseinzadeh-Attar M-J, Sahebkar A, Ranjbar SH, Najafi A, Hosseini S, et al. Therapeutic effects of supplementation with Curcuminoids in critically ill patients receiving enteral nutrition: a randomized controlled trial protocol. Journal of Diabetes & Metabolic Disorders. 2020:1-6. | | | |

**ج) ملاحظات اخلاقی**

| **مطالعات حیوانی** |
| --- |
| **اگر این مطالعه نیازمند استفاده از حیوانات آزمایشگاهی است، گونه و تعداد مورد نیاز را ذکر کنید و توضیح دهید و چه ملاحظاتی را برای رعایت ایمنی و حقوق آنان در نظر می­گیرید.**  در این مطالعه کاربرد ندارد. |
| **بافت­ها یا نمونه­های انسانی** |
| **گر در این مطالعه، از بافت یا نمونه­های مشتق از انسان استفاده می­شود، نوع و تعداد نمونه­ها و ملاحظات اخلاقی مربوطه را بیان کنید.**  در این مطالعه کاربرد ندارد. |
| **آزمودنی­های انسانی** |
| **اگر در این مطالعه هر نوع مداخله­ای بر روی انسان­ها انجام می­شود، تشریح کنید و ملاحظات اخلاقی برای رعایت حقوق آن­ها را تبیین کنید. همچنین تشریح نمایید که چه تدابیری برای حفاظت از آزمودنی‌ها و حفظ ایمنی آنها در طول مطالعه اتخاذ می‌کنید و یک نمونه از فرم رضایت آگاهانه را پیوست نمایید.**  در این مطالعه پس از ارائه توضیحات مکتوب و شفاهی درباره اهداف و روش اجرای پژوهش، از تمامی افراد در صورت تمایل به همکاری در مطالعه، رضایت نامه آگاهانه کتبی(پیوست 1) اخذ خواهد شد. تمامی شرکت کنندگان اجازه خواهند داشت تا هر زمان که خواستند از مطالعه خارج شوند. اطلاعات افراد نیز به صورت محرمانه حفظ می شود. و نتایج آزمایش ها در پایان مطالعه در اختیار افراد قرار می گیرد. کلیه آزمایشات بطور رایگان انجام می شود. |
| **ارسال نمونه­های زیستی** |
| در این مطالعه کاربرد ندارد. |

**د) جدول زمان بندی**

| **عنوان فعالیت** | **مدت زمان اجرا (ماه)** | | | | | | | | | | | | | | | | | | | | | | | | | | | | | |
| --- | --- | --- | --- | --- | --- | --- | --- | --- | --- | --- | --- | --- | --- | --- | --- | --- | --- | --- | --- | --- | --- | --- | --- | --- | --- | --- | --- | --- | --- | --- |
|  | **1** | **2** | **3** | **4** | **5** | **6** | **7** | **8** | **9** | **10** | **11** | **12** | **13** | **14** | **15** | **16** | **17** | **18** | **19** | **20** | **21** | **22** | **23** | **24** | **25** | **26** | **27** | **28** | **29** | **30** |
| \| نگارش پروپوزال \| \| --- \| | * |  |  |  |  |  |  |  |  |  |  |  |  |  |  |  |  |  |  |  |  |  |  |  |  |  |  |  |  |  |
| تصویب پروپوزال |  | * | * |  |  |  |  |  |  |  |  |  |  |  |  |  |  |  |  |  |  |  |  |  |  |  |  |  |  |  |
| اجرای عملی |  |  |  | * | * | * |  |  |  |  |  |  |  |  |  |  |  |  |  |  |  |  |  |  |  |  |  |  |  |  |
| ورود داده ها |  |  |  |  |  | * | * | * |  |  |  |  |  |  |  |  |  |  |  |  |  |  |  |  |  |  |  |  |  |  |
| تجزیه و تحلیل داده ها |  |  |  |  |  |  |  | * | * | * | * |  |  |  |  |  |  |  |  |  |  |  |  |  |  |  |  |  |  |  |
| نگارش مقاله و گزارش دهی نهایی |  |  |  |  |  |  |  |  |  |  |  | * | * | * | * | * | * | * |  |  |  |  |  |  |  |  |  |  |  |  |

**کل زمان اجرای مطالعه (ماه):** 18 ماه **تاریخ تقریبی شروع مطالعه: 10**/99

**هـ) پیش بینی بودجه**

| **کل بودجه طرح (ریال):** | **100000000** |
| --- | --- |

| **اگر برای تمام یا بخشی از این طرح از سازمان دیگری تامین اعتبار شده است، مبلغ و سازمان را ذکر فرمایید.** |
| --- |
|  |

| **سرفصل هزینه­ها برای بودجه درخواستی از دانشگاه** | **مبلغ (ریال)** |
| --- | --- |
| **پرسنلی** | 5800000 |
| **آزمایش­ها و خدمات تخصصی** | 71000000 |
| **مواد مصرفی** (کورکومین، پلاسبو، قوطی، برچسب) | 18200000 |
| **دستگاه­ها و تجهیزات غیر مصرفی** | 0 |
| **مسافرت** | 2000000 |
| **سایر هزینه­ها (با ذکر موضوع هزینه)** | **6 صفحه* 100 سری کپی* هر صفحه 500 تومن**  **کل: 3000000** |
| **جمع بودجه درخواستی از دانشگاه** | 100000000 |

*** جزئیات جدول هزینه­ها در سامانه پژوهشیار به صورت کامل و منطبق بر سرجمع­های بالا تکمیل گردد.**

**هزینه ها**

| **مورد** | **هزینه هر بار به تومان** | **تعداد** | **هزینه کل به تومان** |
| --- | --- | --- | --- |
| **CRP** | **14000** | **100** | **1400000** |
| **ESR** | **7000** | **100** | **700000** |
| **ALT** | **6000** | **100** | **600000** |
| **AST** | **6000** | **100** | **600000** |
| **LDH** | **7000** | **100** | **700000** |
| **BUN** | **6000** | **100** | **600000** |
| **Creatinine** | **6000** | **100** | **600000** |
| **قند خون** | **6000** | **100** | **600000** |
| **آلبومین** | **9000** | **100** | **900000** |
| **CBC** | **4000** | **100** | **400000** |
| **کورکومین پیپرین** | **یک کپسول 500 میلی گرمی 2200 تومان** | **700** | **1540000** |
| **پلاسبو** | **یک کپسول 400 تومان** | **700** | **280000** |
| **کپی پرسش نامه ها** | **6 صفحه* 100 سری کپی* هر صفحه 500 تومن** |  | **300000** |
| **هزینه مسافرت** | **دانشگاه- بیمارستان الزهرا (س) رفت و برگشت در مجموع 10000 تومان** | **20** | **200000** |
| **جمع کل** | **10000000 تومان** | | |

**و) تیم پژوهشی**

| **مجری اصلی** | | | |
| --- | --- | --- | --- |
| **رشته و مدرک تحصیلی:** دکتری تخصصی تغذیه | | **نام و نام خانوادگی:** غلامرضا عسگری | |
| **سازمان متبوع:** دانشگاه علوم پزشکی اصفهان | | **شغل فعلی:** دانشیار | |
| **لاین تحقیقاتی:** تغذیه و التهاب | | | |
| **نقشه پژوهشی و سوابق تحقیقاتی مرتبط با این طرح:**  اینجانب پزشک متخصص تغذیه می باشم که حدود 23 سال سابقه کار در دانشگاه علوم پزشکی اصفهان را دارا می باشم. طراحی و اجرای مطالعات کارآزمایی بالینی بصورت پایان نامه دانشجویان کارشناسی ارشد و دکتری و طرح های پژوهشی در زمینه تغذیه و رژیم درمانی و چاپ مقالات متعدد در این زمینه از جمله سوابق اینجانب بوده. همچنین با توجه به انجام طرح های متعدد در زمینه بیماری های غیر واگیر صلاحیت اجرای این طرح را دارم. با توجه به ارتباط نزدیک اکثر این بیماری ها با التهاب و نقش بسیار موثر رژیم غذایی در ایجاد، افزایش یا کاهش التهاب، در این زمینه مطالعات متعددی از جمله چند پایان نامه و طرح تحقیقاتی در حال اجرا دارم. اینجانب تاکنون مطالعات زیادی در زمینه بیماری های مزمن از جمله چاقی، دیابت و کبد چرب داشته ام. با توجه به اثرات کورکومین بر روی بیماری های ویروسی در مطالعات پایه هم چنین اثرات مطلوب آن بر روی بیماری های ریوی، بنظر می رسد این ماده گیاهی اثرات مطلوبی بر روی ویورس کرونا خواهد داشت. لذا بر آن شدیم تا با یک تیم تحقیقاتی جامع به بررسی اثرات کورکومین بر روی بیماران کرونا بپردازیم. | | | |
| **ISI Impact factor** | **مقالات قبلی مجری مرتبط با موضوع پروژه (حداکثر ده مورد و به ترتیب اهمیت)** | |  |
| 0.8 | Arab A, Askari G, Golshiri P, Feizi A, Hekmatnia A, Iraj B, et al. The effect of a lifestyle modification education on adiposity measures in overweight and obese nonalcoholic fatty liver disease patients. International journal of preventive medicine. 2017;8. | | 1 |
| 1.1 | [Akbarian S-A, Asgary S, Feizi A, Iraj B, Askari G. Comparative study on the effect of Plantago psyllium and Ocimum basilicum seeds on anthropometric measures in nonalcoholic fatty liver patients. International journal of preventive medicine. 2016;7..](https://www.ncbi.nlm.nih.gov/pubmed/27761216) | | 2 |
| 1.4 | Zolfaghari H, Askari G, Siassi F, Feizi A, Sotoudeh G. Intake of nutrients, fiber, and sugar in patients with nonalcoholic fatty liver disease in comparison to healthy individuals. International journal of preventive medicine. 2016;7. | | 3 |
| 1.1 | Rahmani S, Asgary S, Askari G, Keshvari M, Hatamipour M, Feizi A, et al. Treatment of non‐alcoholic fatty liver disease with curcumin: A randomized placebo‐controlled trial. Phytotherapy Research. 2016;30(9):1540-8. | | 4 |
| 3.6 | [Asgharian A, Askari G, Esmailzade A, Feizi A, Mohammadi V. The effect of symbiotic supplementation on liver enzymes, C-reactive protein and ultrasound findings in patients with non-alcoholic fatty liver disease: a clinical trial. International journal of preventive medicine. 2016;7.al.](https://www.ncbi.nlm.nih.gov/pubmed/27076897) | | 5 |
| 1.1 | Foroughi M, Maghsoudi Z, Khayyatzadeh S, Ghiasvand R, Askari G, Iraj B. Relationship between non-alcoholic fatty liver disease and inflammation in patients with non-alcoholic fatty liver. Advanced biomedical research. 2016;5. | | 6 |
| 2.4 | Foroughi M, Maghsoudi Z, Askari G. The effect of vitamin D supplementation on blood sugar and different indices of insulin resistance in patients with non-alcoholic fatty liver disease (NAFLD). Iranian journal of nursing and midwifery research. 2016;21(1):100. | | 7 |
| 1.8 | Soleimani D, Paknahad Z, Askari G, Iraj B, Feizi A. Effect of garlic powder consumption on body composition in patients with nonalcoholic fatty liver disease: A randomized, double-blind, placebo-controlled trial. Advanced biomedical research. 2016;5.l | | 8 |
| 1.4 | Pezeshki A, Safi S, Feizi A, Askari G, Karami F. The effect of green tea extract supplementation on liver enzymes in patients with nonalcoholic fatty liver disease. International journal of preventive medicine. 2016;7. | | 9 |
| 1.2 | Foroughi M, Maghsoudi Z, Ghiasvand R, Iraj B, Askari G. Effect of vitamin D supplementation on C-reactive protein in patients with nonalcoholic fatty liver. International journal of preventive medicine. 2014;5(8):969. | | 10 |

| **مجری دوم** | | | |
| --- | --- | --- | --- |
| **رشته و مدرک تحصیلی:** فوق تخصص آی سی یو و بیهوشی | | **نام و نام خانوادگی:** بایک علی کیایی | |
| **سازمان متبوع:** دانشگاه علوم پزشکی اصفهان | | **شغل فعلی:**  دانشیار | |
| **لاین تحقیقاتی:** آی سی یو و بیهوشی | | | |
| **نقشه پژوهشی و سوابق تحقیقاتی مرتبط با این طرح:**  اینجانب تاکنون مطالعات زیادی در زمینه تغذیه بیماران بد حال بستری در ICU داشته ام. با توجه به شیوع بالای کرونا در این روزها و مشکلات فراوان این بیماران در ICU بخصوص با توجه به التهاب بالا در این افراد و عدم وجود درمان مناسب، تصمیم گرفتیم تا با یک تیم تحقیقاتی جامعه به بررسی اثر مکمل کورکومین پیپرین در بیماران مبتلا به کرونا بستری در آی سی یو داشته باشیم. بنظر می رسد نتایج این طرح برای اولین بار در دنیا میتواند موجب بهبود وضعیت بیماران بستری در ICU گردد. در همین راستا مطالعه مرور سیستماتیک اخیر که در مجله معتبر فیتوتراپی ریسرچ منتشر شده بیانگر اثرات مطلوب کورکومین بر روی سپسیس در مطالعات حیوانی و مولکولار بوده است. با توجه به عدم وجود کارآزمایی بالینی در این زمینه، این طرح بعنوان اولین طرح تحقیقاتی در این زمینه میتواند نتایج حائز اهمیتی ارائه نماید. | | | |
| **ISI Impact factor** | **مقالات قبلی مجری مرتبط با موضوع پروژه (حداکثر ده مورد و به ترتیب اهمیت)** | |  |
| 1.467 | Ghiasi F, Moghadam KG, Alikiaii B, Sadrzadeh S, Farajzadegan Z. The prognostic value of rapid shallow breathing index and physiologic dead space for weaning success in intensive care unit patients under mechanical ventilation. Journal of research in medical sciences: the official journal of Isfahan University of Medical Sciences. 2019;24. | | 1 |
| Scopus | ALIKIAII B, AGHADAVOUDI O, EMAMI N. Evaluating antibiotic resistance pattern of ventilator-associated pneumonia in intensive care units of Alzahra Hospital, Isfahan University of Medical Sciences, Iran. Journal of Isfahan Medical School. 2016;34(399), pp. 1083-1089 | | 2 |
| Scopus | ALIKIAII B, HASHEMI ST, MANTEGHI F. Evaluation of platelet elevation after injection in patients over 40 years of age admitted hospital; a retrospective study. Journal of Isfahan Medical School. 2017;35(451), pp. 1438-1443 | | 3 |
| Scopus | MORADI FD, NAGHIBI K, Alikiaei B, Mashayekhi Z. Comparison of the effects of intravenous phenylephrine and ephedrine in treatment of hypotension after spinal anesthesia in orthopedic surgery. Journal of Babol University of Medical Sciences. 2016; 18 (6) 21-27. | | 4 |
| Scopus | ALIKIAII B, KARAMI S, HASHEMI ST, MOUSAVI S. Comparison of the effect of different doses of selenium on maximum inspiratory pressure among patients receiving blood transfusion in intensive care units. Journal of Isfahan Medical School. 2017;35(421), 186-192 | | 5 |
| Scopus | MORADI FD, NAGHIBI K, TAHERI S, ALIKIAII B, RAHIMI VM. Effects of age and gender on acute postoperative pain after cataract surgery under topical anesthesia and sedation. Journal of Isfahan Medical School. . 2017;34(414), 1627-1633 | | 6 |
| Scopus | MORADI FD, AKRAMI F, NAGHIBI K, ALIKIAII B, NAZEMORROAYA B. The effect of age and sex on postoperative pain after deep vitrectomy. Journal of Isfahan Medical School. 2017;34(415), 1660-1665 | | 7 |
| Scopus | Alikiaii B, Kashefi P, Abbasi S, Askari-Barzani E. Comparison of serum level of magnesium in patients received pantoprazole or ranitidine in intensive care unit. Journal of Isfahan Medical School. 2017;35(432):615-21. | | 8 |
| Scopus | ALIKIAII B, ABBASI S, MORADI FD, HASHEMI ST, GHASEMI K. Diagnostic values of troponin I and T for prediction of mortality in patients hospitalized in intensive care unit and need hemodialysis in Alzahra hospital, Isfahan, Iran, during 2015-16. Journal of Isfahan Medical School. 2017; 35(442), 1000-1005 | | 9 |

| **مجری سوم** | | | |
| --- | --- | --- | --- |
| **رشته و مدرک تحصیلی:** دکتری تخصصی (PhD) تغذیه | | **نام و نام خانوادگی:** دکترمحمد باقرنیا | |
| **سازمان متبوع:** دانشگاه علوم پزشکی اصفهان | | **شغل فعلی:** استادیار تغذیه | |
| **لاین تحقیقاتی: تغذیه و بیماری های غیر واگیر** | | | |
| **نقشه پژوهشی و سوابق تحقیقاتی مرتبط با این طرح:**  اینجانب تاکنون مطالعات زیادی در زمینه بیماری های مزمن از جمله چاقی، دیابت و کبد چرب داشته ام. اینجانب اخیرا با یک تیم تحقیقاتی جامع، تحقیقات زیادی در این زمینه طرح ریزی کرده ام. از جمله چند مطالعه درباره اثرات کورکومین (همراه با پیپرین یا فیتوزومال کورکومین با کولین یا سرین) بر روی بیماری های مزمن به خصوص کبد چرب غیر الکلی. با توجه به اهمیت موضوع و اثرات مطلوب کورکومین بر روی بیماری های ویروسی و ریوی در مطالعات پایه، بنظر می رسد انجام مطالعه حاضر باعث بهبود شرایط بیماران می گردد. | | | |
| **ISI Impact factor** | **مقالات قبلی مجری مرتبط با موضوع پروژه (حداکثر ده مورد و به ترتیب اهمیت)** | |  |
| 10.39 | Bagherniya M, Butler AE, Barreto GE, Sahebkar A. The effect of fasting or calorie restriction on autophagy induction: A review of the literature. Ageing research reviews. 2018;47:183-97. | | 1 |
| 5.5 | Bagherniya M, Nobili V, Blesso CN, Sahebkar A. Medicinal plants and bioactive natural compounds in the treatment of non-alcoholic fatty liver disease: A clinical review. Pharmacological research. 2018;130:213-40. | | 2 |
| 3.08 | Khayyatzadeh SS, Bagherniya M, Fazeli M, Khorasanchi Z, Bidokhti MS, Ahmadinejad M, et al. A Western dietary pattern is associated with elevated level of high sensitive C-reactive protein among adolescent girls. European journal of clinical investigation. 2018;48(4). | | 3 |
| 1.89 | Bagherniya M, Khayyatzadeh SS, Heidari Bakavoli AR, Ferns GA, Ebrahimi M, Safarian M, et al. Serum high-sensitive C-reactive protein is associated with dietary intakes in diabetic patients with and without hypertension: a cross-sectional study. Annals of clinical biochemistry. 2018;55(4):422-9. | | 4 |
| 1.3 | Bagherniya M, Taghipour A, Sharma M, Sahebkar A, Contento IR, Keshavarz SA, et al. Obesity intervention programs among adolescents using social cognitive theory: a systematic literature review. Health education research. 2018;33(1):26-39 | | 5 |
| ISI | Bagherniya M, Khayyatzadeh SS, Avan A, Safarian M, Nematy M, Ferns GA, et al. Metabolic syndrome and its components are related to psychological disorders: A population based study. Diabetes & metabolic syndrome. 2017;11 Suppl 2:S561-s6 | | 6 |
| 3.2 | Khayyatzadeh SS, Bagherniya M, Abdollahi Z, Ferns GA, Ghayour-Mobarhan M. What is the best solution to manage vitamin D deficiency? IUBMB life. 2019;71(9):1190-1. | | 7 |
| 2.66 | Rostami H, Khayyatzadeh SS, Tavakoli H, Bagherniya M, Mirmousavi SJ, Farahmand SK, et al. The relationship between adherence to a Dietary Approach to Stop Hypertension (DASH) dietary pattern and insomnia. BMC psychiatry. 2019;19(1):234 | | 8 |
| 1.46 | Shafi H, Dorosty Motlagh AR, Bagherniya M, Daeezadeh A, Safarian M. The Association of Household Food Insecurity and the Risk of Calcium Oxalate Stones. Urology journal. 2017;14(5):4094-5000. | | 9 |
| 3.59 | Tavakoli H, Rostami H, Avan A, Bagherniya M, Ferns GA, Khayyatzadeh SS, et al. High dose vitamin D supplementation is associated with an improvement in serum markers of liver function. BioFactors (Oxford, England). 2019;45(3):335-42. | | 10 |

| **مجری چهارم** | | | |
| --- | --- | --- | --- |
| **رشته و مدرک تحصیلی:** فوق تخصص غدد | | **نام و نام خانوادگی:** دکتر بیژن ایرج | |
| **سازمان متبوع:** دانشگاه علوم پزشکی اصفهان | | **شغل فعلی:** دانشیار دانشگاه | |
| **نقشه پژوهشی و سوابق تحقیقاتی مرتبط با این طرح:**  با توجه به اهمیت موضوع و اثرات مطلوب کورکومین بر روی بیماری های ویروسی و ریوی در مطالعات پایه، بنظر می رسد انجام مطالعه حاضر باعث بهبود شرایط بیماران می گردد. | | | |
| **ISI Impact factor** | **مقالات قبلی مجری مرتبط با موضوع پروژه (حداکثر ده مورد و به ترتیب اهمیت)** | |  |
| 6.402 | Jafari T, Faghihimani E, Feizi A, Iraj B, Javanmard SH, Esmaillzadeh A, et al. Effects of vitamin D-fortified low fat yogurt on glycemic status, anthropometric indexes, inflammation, and bone turnover in diabetic postmenopausal women: A randomised controlled clinical trial. Clin Nutr. 2016;35(1):67-76. | | 1 |
| 2.287 | Salari Moghaddam A, Entezari MH, Iraj B, Askari G, Sharifi Zahabi E, Maracy MR. The effects of soy bean flour enriched bread intake on anthropometric indices and blood pressure in type 2 diabetic women: a crossover randomized controlled clinical trial. Int J Endocrinol. 2014;2014:240760. | | 2 |
| 2.638 | Vatandoost N, Amini M, Iraj B, Momenzadeh S, Salehi R. Dysregulated miR-103 and miR-143 expression in peripheral blood mononuclear cells from induced prediabetes and type 2 diabetes rats. Gene. 2015;572(1):95-100. | | 3 |
| 0.834 | Tahmasebi A, Amin MM, Poursafa P, Iraj B, Sadeghiyan H, Kelishadi R, et al. Association of geographical distribution of air quality index and type 2 diabetes mellitus in Isfahan, Iran. Pak J Med Sci. 2015;31(2):369-73. | | 4 |
| 2.895 | Askari G, Iraj B, Salehi-Abargouei A, Fallah AA, Jafari T. The association between serum selenium and gestational diabetes mellitus: a systematic review and meta-analysis. J Trace Elem Med Biol. 2015;29:195-201. | | 5 |
| 1.521 | Taheri N, Iraj B, Amini M, Amini P, Aminorroaya A. Cardiovascular risk factors in relatives of type 2 diabetics with normal glucose tolerance test and elevated one-hour plasma glucose. Endokrynol Pol. 2010;61(4):359-63. | | 6 |
| 1.467 | Vanaie A, Shahidi S, Iraj B, Siadat ZD, Kabirzade M, Shakiba F, et al. Curcumin as a major active component of turmeric attenuates proteinuria in patients with overt diabetic nephropathy. J Res Med Sci. 2019;24:77. | | 7 |
| 1.467 | Gholi Z, Heidari-Beni M, Feizi A, Iraj B, Askari G. The characteristics of pre-diabetic patients associated with body composition and cardiovascular disease risk factors in the Iranian population. J Res Med Sci. 2016;21:20. | | 8 |
| 1.467 | Kachuei A, Amini M, Sebghatollahi V, Feizi A, Hamedani P, Iraj B. Effect of Helicobacter pylori eradication on insulin resistance among prediabetic patients: A pilot study and single-blind randomized controlled clinical trial. J Res Med Sci. 2016;21:8. | | 9 |
| 1.938 | Fatemi A, Iraj B, Barzanian J, Maracy M, Smiley A. Musculoskeletal manifestations in diabetic versus prediabetic patients. Int J Rheum Dis. 2015;18(7):791-9. | | 10 |

| **همکار طرح** | | | |
| --- | --- | --- | --- |
| **رشته و مدرک تحصیلی: آمار زیستی و اپیدمیولوژی** | | **نام و نام خانوادگی: آوات فیضی** | |
| **سازمان متبوع: دانشگاه علوم پزشکی اصفهان** | | **شغل فعلی: استاد دانشگاه** | |
| **لاین تحقیقاتی: آمار زیستی و اپیدمیولوژی** | | | |
| **نحوه مشارکت در این طرح و توانمندی­های مرتبط**  اینجانب بعنوان مشاور آماری در طرح حاضر که یک طرح کاربردیست دارم. امیدوارم نتایج این طرح بتواند مشکلات بیماران کرونای بستری در آی سی یو را کاهش دهد. با توجه به سوابق فراوان اینجانب در همکاری کارهای کارآزمایی بالینی، اینجانب صلاحیت شرکت در این طرح را دارا هستم. | | | |
| **ISI Impact factor** | **مقالات قبلی همکار مرتبط با موضوع پروژه (حداکثر پنج مورد و به ترتیب اهمیت)** | |  |
| 2.29 | Azimi P, Ghiasvand R, Feizi A, Hosseinzadeh J, Bahreynian M, Hariri M, et al. Effect of cinnamon, cardamom, saffron and ginger consumption on blood pressure and a marker of endothelial function in patients with type 2 diabetes mellitus: A randomized controlled clinical trial. Blood pressure. 2016;25(3):133-40. | | 1 |
| 6.42 | Jafari T, Faghihimani E, Feizi A, Iraj B, Javanmard SH, Esmaillzadeh A, et al. Effects of vitamin D-fortified low fat yogurt on glycemic status, anthropometric indexes, inflammation, and bone turnover in diabetic postmenopausal women: A randomised controlled clinical trial. Clinical nutrition. 2016;35(1):67-76. | | 2 |
| 76.33.76 | Rahmani S, Asgary S, Askari G, Keshvari M, Hatamipour M, Feizi A, et al. Treatment of non‐alcoholic fatty liver disease with curcumin: A randomized placebo‐controlled trial. Phytotherapy Research. 2016;30(9):1540-8. | | 3 |
| 3.34 | Asemi Z, Saneei P, Sabihi S-S, Feizi A, Esmaillzadeh A. Total, dietary, and supplemental calcium intake and mortality from all-causes, cardiovascular disease, and cancer: A meta-analysis of observational studies. Nutrition, Metabolism and Cardiovascular Diseases. 2015;25(7):623-34. | | 4 |
| 4.49 | Salehi-Abargouei A, Esmaillzadeh A, Azadbakht L, Keshteli AH, Feizi A, Feinle-Bisset C, et al. Nutrient patterns and their relation to general and abdominal obesity in Iranian adults: findings from the SEPAHAN study. European journal of nutrition. 2016;55(2):505-18. | | 5 |


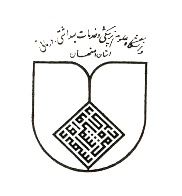
**فرم رضايت نامه آگاهانه شرکت در طرح تحقیقاتی**

| **آقاي/خانم محترم**  بدين وسيله از شما جهت شركت در پژوهش زیر دعوت به عمل مي‌آيد. اطلاعات مربوط به این پژوهش، در این برگه خدمتتان ارائه شده است و **شما برای شرکت یا عدم شرکت در این پژوهش آزاد هستید**. **شما مجبور به تصميم گيري فوري نيستيد** و برای تصميم گيري در اين باره، مي‌توانيد سوالات خود را از تيم پژوهشي بپرسيد و با هر فردي که مایل باشید مشورت نماييد. قبل از امضاي اين رضايت نامه، مطمئن شويد كه متوجه تمامي اطلاعات اين فرم شده‌ايد و به تمام سوالات شما پاسخ داده شده است.  **نام مجري پژوهش** |
| --- |

| **عنوان طرح پژوهشی** | بررسی اثر مکملیاری با کورکومین-پیپرین در بیماران مبتلا به ویروس کرونا بستری در بخش مراقبت های ویژه (ICU): یک مطالعه کارآزمایی بالینی دو سو کور |
| --- | --- |
| **نام مجری یا مجریان** | دکتر غلامرضا عسکری، دکتر بابک علی کیایی |
| **دانشکده یا واحد مربوطه** | دانشکده تغذیه و علوم غذایی، گروه تغذیه جامعه |
| **معرفی پژوهش** | کورکومین به عنوان یک ماده ی موثر در بهبود بسیاری از بیماری های مزمن و برای کاهش التهاب و عوارض بیماری ها نام برده شده است، همچین در مطالعات پایه دارای اثرات مطلوب بر روی بیماری آنفولانزا بوده است. کورکومین همان عصاره یا ماده ی موثره ی زردچوبه (2 تا 5 درصد زردچوبه، کورکومین است. کورکومین عصاره زردچوبه است) می باشد. کورکومین در آب حلالیت کمی دارد و جذب پایینی در دستگاه گوارش دارد. پیپرین عصاره فلفل می باشد که باعث حلالیت بالاتر کورکومین می شود. هدف از این مطالعه بررسی اثر کورکومین-پیپرین بر روی وضعیت سلامتی بیماری ویروسی کرونا در بیماران بستری در ICU می باشد. |
| **مداخله** | بیمار من که بعلت ابتلا به ویروس کرونا در بخش مراقبت های ویژه بستری است بصورت تصادفی در یکی از دو گروه زیر قرار میگیرد: گروه مداخله مکمل کورکومین/پیپرین 500 میلیگرمی (روزانه سه کپسول؛ در مجموع 1500 میلیگرم کورکومین و 15 میلیگرم پیپرین) و گروه شاهد دارونما (مالتودکسترین، 500 میلی گرم روزانه سه کپسول در مجموع 1500 میلیگرم مالتودکسترین، این ماده دارونماست و بی اثر است) به مدت 2 هفته (14 روز) را دریافت خواهند کرد. |
| **نحو ه ی همکاری شرکت کننده** | بیماری که به علت ابتلا به ویروس کرونا در بخش مراقبت های ویژه بستری است بصورت تصادفی در یکی از دو گروه زیر قرار میگیرد:  1) گروه مداخله مکمل کورکومین/پیپرین 500 میلیگرمی (روزانه سه کپسول؛ در مجموع 1500 میلیگرم کورکومین و 15 میلیگرم پیپرین) به مدت 14 روز  2) گروه شاهد دارونما (مالتودکسترین، 500 میلی گرم روزانه سه کپسول در مجموع 1500 میلیگرم مالتودکسترین، این ماده دارونماست و بی اثر است) به مدت 14 روز  قبل و بعد از مداخله از بیمار 10 سی سی خون گرفته می شود همچنین اطلاعات مربوط به سن،جنس، قد، وزن و بیماری های زمینه ای و اطلاعات مربوط به شدت بیماری بیمار ثبت میگردد. |
| **منافع احتمالي شرکت در اين مطالعه** | مکمل کورکومین-پیپرین استفاده شده در این طرح ممکن است به بهبود وضعیت بیمار من کمک کند. تمامي آزمايشات این طرح به صورت کاملا رايگان انجام خواهد شد. |
| **آسيب‌ها و عوارض احتمالي شرکت در اين مطالعه و نحوه جبران خسارت** | شرکت در این مطالعه بر اساس گزارشات قبلی هیچگونه عوارض و خطرات احتمالی برای افراد ندارد. در صورت وجود حساسیت به مواد گیاهی مانند زردچوبه و فلفل ممکن است مصرف مکمل برای شما عوارضی ایجاد کند. در صورت وجود حساسیت نباید در این طرح مشارکت نمایید. در صورت مشاهده هرگونه عارضه از روش درماني قطع مداخله جهت مداوای عوارض جانبي ناخوشايند استفاده میشود. خونگیری ممکن است با ضعف برای بیمار همراه باشد.  من مي‌دانم كه اگر در حين و بعد از انجام پژوهش، هر مشكل جسمي و روحي که به علت شرکت در اين پژوهش براي من پيش آمد، درمان عوارض و هزينه‌هاي آن و غرامت مربوطه بر عهده مجري خواهد بود. |
| **هزینه** | تمام هزینه های مربوط به این طرح شامل هزینه مکمل ها و دارونما، ویزیت های دوره ای، خونگیری، آزمایشات مربوط به پارامتر بیوشیمیایی، کپی پرسش نامه ها و غیره بعهده مجری طرح می باشد و بیمار و ولی و همراه وی هیچ هزینه ای برای این طرح پرداخت نخواهند کرد. "هزینه تمام مداخلات پژوهشی ذکر شده، به عهده مجری یا مجریان طرح خواهد بود و بیمار هزینه ای پرداخت نخواهد کرد." |
| **روش های جایگزین** | اگر این پژوهش به هر دلیلی خارج از موعد مقرر خاتمه یابد یا به حالت تعلیق درآید، بموقع اطلاع خواهم یافت و درمان مناسب برای بیمار من ادامه خواهد یافت و رها نخواهم شد.  در صورت عدم تمایل به شرکت در مطالعه روش معمول درمانی برای بیمار من ارائه خواهد شد. |
| **محرمانه بودن** | - من مي­دانم كه دست اندر كاران اين پژوهش، كليه اطلاعات مربوط به من را نزد خود به صورت محرمانه نگه‌ داشته و فقط اجازه دارند نتايج كلي و گروهي اين پژوهش را بدون ذکر نام و مشخصات اينجانب منتشر كنند. همچنین من می توانم نتایج بررسی فردی خودم را داشته باشم. - من می­دانم که كميته اخلاق در پژوهش دانشگاه، با هدف نظارت بر رعایت حقوق اينجانب، مي‌تواند به اطلاعات من دسترسي داشته باشد. |
| **پاسخگویی به پرسش ها** | آقاي دکتر غلامرضا عسکری جهت پاسخگويي به اينجانب معرفي شد و به من گفته شد تا هر وقت مشكلي يا سوالي در رابطه با شركت در پژوهش مذكور پيش آمد با ايشان در ميان بگذارم و راهنمايي بخواهم. آدرس و شماره تلفن ثابت و همراه ايشان به شرح زیر به من ارائه شد:  **آدرس:** اصفهان ، خیابان هزارجریب ، دانشگاه علوم پزشکی اصفهان،دانشکده تغذیه و علوم غذایی  **تلفن ثابت: 03137923171**  **تلفن همراه: 09132663418** |
| **حق نپذیرفتن، حق انصراف و حق شکایت و اعتراض** | 1- من مي­دانم که شرکت من در اين پژوهش کاملاً داوطلبانه است و مجبور به شرکت در اين پژوهش نيستم.  2- من میدانم که دراین پژوهش، بطور تصادفی ممکن است در گروه مورد یا گروه کنترل قراربگیرم  3-به من اطمينان داده شد که اگر حاضر به شركت در اين پژوهش نباشم، از مراقبت‌هاي معمول تشخيصي و درماني محروم نخواهم شد و رابطه درماني من با مركز درماني و پزشك معالجم دچار اشكال نمي‌شود.  4- من مي‌دانم كه حتي پس از موافقت با شركت در پژوهش، مي‌توانم هر وقت كه بخواهم، پس از اطلاع به مجري، از پژوهش خارج شوم و خروج من از پژوهش باعث محرومیت از دریافت خدمات تشخیصی/درمانی معمول برای من نخواهد شد.  5-به من اطمینان داده شد چنانچه تغییری در اجرای پژوهش یا اطلاعات جدیدی در حین اجرا به دست آمد که آگاهی از آن ممکن است تصمیم مرا برای ادامه شرکت در پژوهش تغییر دهد، مجری/مجریان طرح ضمن اطلاع به کمیته اخلاق دانشگاه، الزاما مرا در جریان قرار دهند و مجددا رضایت نامه آگاهانه را کتبا تکمیل نمایم.  6- به من اطمینان داده شد که اگر این پژوهش به هر دلیلی خارج از موعد مقرر خاتمه یابد یا به حالت تعلیق درآید، بموقع اطلاع خواهم یافت و درمان مناسب برای من ادامه خواهد یافت و رها نخواهم شد.  7- من مي­دانم که اگر اشکال يا اعتراضي نسبت به دست اندركاران يا روند پژوهش داشته باشم، مي­توانم با **كميته اخلاق در پژوهش دانشگاه علوم پزشكي اصفهان به آدرس اصفهان، خیابان هزار جریب، دانشگاه علوم پزشکی اصفهان، ساختمان شماره 4 ستادی، طبقه دوم، اتاق 209، دبیرخانه دائمی کمیته اخلاق در پژوهش دانشگاه، تلفن 37923054 (031)** تماس گرفته و مشکل خود را به صورت شفاهی یا کتبی مطرح نمایم. |
| **رضایت نهایی شرکت کننده** | اين فرم "اطلاعات و رضايت آگاهانه" در **دو نسخه** تنظيم شده و پس از امضا، يک نسخه در اختيار من و نسخه ديگر در اختيار مجري قرار خواهد گرفت.  اينجانب، موارد فوق‌الذکر را خواندم و فهميدم و بر اساس آن رضايت آگاهانه خود را براي شركت در اين پژوهش اعلام مي‌کنم**.**  **نام و نام خانوادگی شرکت کننده (یا قیم قانونی وی):**  **امضای شرکت کننده (یا قیم قانونی وی):**  **تاریخ امضا / / شماره موبایل جهت ارتباط:** |
| **تعهد اخلاقی پژوهشگر** | اينجانب ………………، خود را ملزم به اجراي تعهدات مربوط به مجري در مفاد فوق دانسته و متعهد مي‌گردم در تأمين حقوق و ايمني شركت كننده در اين پژوهش تلاش نمايم.  **مهر و امضاي پژوهشگر** |

**پیوست شماره 2 (پرسش نامه اطلاعات پایه و دموگرافیک)**

هو الشافی

**عنوان طرح: بررسی اثر مکملیاری با کورکومین-پیپرین در بیماران مبتلا به ویروس کرونا بستری در بخش مراقبت های ویژهICU) ) : یک مطالعه کارآزمایی بالینی دو سو کور**

**فرم بیماران بستری در آی سی یو**

تاریخ

کد بیمار

مرحله 1 2

**شماره تماس: تاریخ تولد (سن): تاریخ پذیرش:**

**جنس** زن مرد

**وضعیت تاهل** مجرد متاهل

**میزان تحصیلات** کمتر از دیپلم دیپلم لیسانس دکترا و بالاتر

**شغل**  بیکار کارمند آزاد کادر درمانی

**بیماری زمینه ای**

دیابت فشارخون سرطان قلبی-عروقی بیماری تنفس

بیماری کلیوی بیماری کبدی بیماری عصبی هیپرلیپیدمی

**سابقه مصرف دارو** (نام دارو و دوز آن ذکر شود) کورتیکواستروئید

**داروهایی که در حال حاضر دریافت می کند: (در طی درمان تغییری در روند داروهای مصرفی ایجاد شده است).**

**مصرف سیگار یا دخانیات (چند عدد در روز):**

**پیوست شماره 3 (پرسش نامه شدت بیماری)**

**عنوان طرح: بررسی اثر مکملیاری با کورکومین-پیپرین در بیماران مبتلا به ویروس کرونا بستری در بخش مراقبت های ویژهICU) ) : یک مطالعه کارآزمایی بالینی دو سو کور**

**فرم بیماران بستری در آی سی یو**

تاریخ

کد بیمار

مرحله 1 2

**علائم بالینی بیمار**

| **علامت** | **ندارد** | **دارد** | **خیلی خفیف** | **خفیف** | **متوسط** | **شدید** | **خیلی شدید** | **طول مدت (روز)** |
| --- | --- | --- | --- | --- | --- | --- | --- | --- |
| سرفه خشک |  |  |  |  |  |  |  |  |
| سرفه خلط دار |  |  |  |  |  |  |  |  |
| لرز |  |  |  |  |  |  |  |  |
| گلودرد |  |  |  |  |  |  |  |  |
| علائم کوریزا(آبریزش،اشک ریزش،گرفتگی بینی) |  |  |  |  |  |  |  |  |
| ضعف |  |  |  |  |  |  |  |  |
| دردعضلانی |  |  |  |  |  |  |  |  |
| سردرد |  |  |  |  |  |  |  |  |
| تنگی نفس |  |  |  |  |  |  |  |  |

**علائم حیاتی**

| **Level of consciousness(GCS)** | | | |
| --- | --- | --- | --- |
| **Pulse Rate** | | | |
| **Respiratory Rate** | | | |
| **Blood Pressure** | | | |
| **Axillary** | **Oral** | **Temperature** | |
| **O2 Saturation** | | | |
| **PaO2/FiO2** | | | |
| **Urin Output** | | | |

**علائم بیوشیمیایی بیمار (موارد ستاره دار الزامیست)**

| **CBC** | | | | | | | |
| --- | --- | --- | --- | --- | --- | --- | --- |
| **Plt*** | **RDW*** | **MCHC*** | **MCH*** | **Hb*** | **Lymph*** | **Nut*** | **WBC *** |
| **Albumin*** | | | | TG | | Procalcitonin | |
| **Hs-CRP*** | | | | Total Cholesterol | | D-Dimer | |
| **ESR*** | | | | LDL | | Troponin | |
| **Creatinine*** | | | | HDL | | Bil (T) | |
| **BUN*** | | | | FBS | | PT | |
| **AST*** | | | | **SOD*(Superoxide dismutase)** | | Ferritin | |
| **ALT*** | | | | **TAC* (Total antioxidant capacity)** | | ABG or VBG | |
| **LDH*** | | | | **MDA*(Malondialdehyde)** | | Total protein | |
| PO2(ABG) | | HCO3 | | PCO2 | | Ph | |

**علائم تصویربرداری بیمار CT chest**

آیا دارای نمای تایید کننده پنومونی ویروسی در سی تی اسکن می باشد ؟(Bilateral, Peripheral, Lower Lobes Opacity)

بله خیر

Multi lobular opacities:

Yes No

**معاینات تغذیه ای**

| وزن (تخمینی) |
| --- |
| قد (تخمینی) |
| BMI (تخمینی) |
| دور بازو |

**بیمار گاواژ خود را براحتی دریافت می کند؟**

**بیمار عدم تحمل گاواژ دارد؟ (بالای 250 سی سی)**

**مکمل مصرفی باعث ایجاد عوارض نامطلوبی در بیمار گردید؟**

**اگر پاسخ مثبت است چه عارضه یا عوارضی؟**

**چند بار این اتفاق افتاد؟**

1. Coronaviruses [↑](#footnote-ref-1)
2. Middle East Respiratory Syndrome (MERS-CoV) [↑](#footnote-ref-2)
3. Severe Acute Respiratory Syndrome (SARS-CoV) [↑](#footnote-ref-3)
4. novel coronavirus (nCoV) [↑](#footnote-ref-4)
5. Coronaviruses [↑](#footnote-ref-5)
6. Middle East Respiratory Syndrome (MERS-CoV) [↑](#footnote-ref-6)
7. Severe Acute Respiratory Syndrome (SARS-CoV) [↑](#footnote-ref-7)
8. novel coronavirus (nCoV) [↑](#footnote-ref-8)
9. glucuronidase enzyme [↑](#footnote-ref-9)
